# Natural Genetic Variation in Selected Populations of *Arabidopsis thaliana* Is Associated with Ionomic Differences

**DOI:** 10.1371/journal.pone.0011081

**Published:** 2010-06-14

**Authors:** Elizabeth Buescher, Tilman Achberger, Idris Amusan, Anthony Giannini, Cherie Ochsenfeld, Ana Rus, Brett Lahner, Owen Hoekenga, Elena Yakubova, Jeffrey F. Harper, Mary Lou Guerinot, Min Zhang, David E. Salt, Ivan R. Baxter

**Affiliations:** 1 Department of Agronomy, Purdue University, West Lafayette, Indiana, United States of America; 2 Department of Statistics, Purdue University, West Lafayette, Indiana, United States of America; 3 Department of Animal Sciences, Purdue University, West Lafayette, Indiana, United States of America; 4 Center for Plant Environmental Stress Physiology, Purdue University, West Lafayette, Indiana, United States of America; 5 Robert W. Holley Center for Agriculture and Health, United States Department of Agriculture-Agricultural Research Service, Cornell University, Ithaca, New York, United States of America; 6 University of Nevada, Reno, Reno, Nevada, United States of America; 7 Department of Biological Sciences, Dartmouth College, Hanover, New Hampshire, United States of America; 8 Plant Genetics Research Unit, United States Department of Agriculture-Agricultural Research Service, Donald Danforth Plant Sciences Center, St. Louis, Missouri, United States of America; University of Umeå, Sweden

## Abstract

Controlling elemental composition is critical for plant growth and development as well as the nutrition of humans who utilize plants for food. Uncovering the genetic architecture underlying mineral ion homeostasis in plants is a critical first step towards understanding the biochemical networks that regulate a plant's elemental composition (ionome). Natural accessions of *Arabidopsis thaliana* provide a rich source of genetic diversity that leads to phenotypic differences. We analyzed the concentrations of 17 different elements in 12 *A. thaliana* accessions and three recombinant inbred line (RIL) populations grown in several different environments using high-throughput inductively coupled plasma- mass spectroscopy (ICP-MS). Significant differences were detected between the accessions for most elements and we identified over a hundred QTLs for elemental accumulation in the RIL populations. Altering the environment the plants were grown in had a strong effect on the correlations between different elements and the QTLs controlling elemental accumulation. All ionomic data presented is publicly available at www.ionomicshub.org.

## Introduction

Genetic variation occurring among and within natural populations of *Arabidopsis thaliana* can be used as a tool for gene discovery [1]–[3]. *A. thaliana* has a wide-geographic distribution, producing a large and diverse group of natural populations, many of which have been collected as accessions that are curated by the Arabidopsis Biological Resource Center (ABRC). Considerable variation for such traits as resistance to biotic and abiotic stress, development, and metabolism has been described (for recent reviews see [3], [4]). Observed variation between accessions can be qualitative, defined by phenotypic distributions that fall into discrete classes, and is caused by one or two major loci. Variation can also be quantitative, defined by a continuous phenotypic distribution, caused by the combined effect of multiple loci. Experimental population size plays a major role in the threshold for detection of loci. Small populations are useful for identifying loci if a trait is controlled by a few loci with large phenotypic effect; however, more complex traits controlled by multiple loci with relatively small phenotypic effect will require large experimental populations.

By using immortalized mapping populations known as recombinant inbred lines (RIL), derived from a variety of accessions, quantitative trait loci (QTL) have been identified for numerous important traits related to the ionome [5]. These include phosphate accumulation in seed and shoot [6], nitrogen (N) use efficiency [7], [8], aluminum (Al) resistance [9]–[11], shoot cesium (Cs) accumulation [12] and shoot selenate accumulation [13], [14]. Once QTLs for traits of interest have been identified, the genomic tools available for *A. thaliana* can be used to locate the genes that underlie these QTLs and thus describe the traits at a molecular level (for a review see [15]). Such an approach was recently taken in our laboratories to identify At*HKT1*, which encodes a Na-transporter, as the gene responsible for a QTL that controls elevated Na in two distinct natural accessions Tsu-1 and Ts-1 [16], At*MOT1*, a putative Mo transporter as the gene responsible for an ∼80% decrease in Mo accumulation in 7 accessions [17] and *FPN2*, an Fe and Co transporter as the gene responsible for increased Co in 6 accessions [18].

Although many studies have examined the genetic basis that controls the accumulation of single elements in a RIL population, only a small number [19]–[21] have examined multiple elements in multiple populations to investigate the genetic architecture of the ionome. We have previously shown that physiological responses to low Fe and P growth conditions cause robust 5 and 6 element signatures, respectively, in *A. thaliana* [22], demonstrating that single element examinations of a population cannot give an accurate picture of the plant ionome. Genetic variation in the response to these and other environmental changes are likely to alter these element- to- element relationships, leading to gene×environment×ionome interactions. In this study, we analyzed a small diversity panel of natural accessions and three RIL populations. We show that significant variation exists in the shoot and the seed ionomes of 12 *A. thaliana* accessions across different environments. The RIL populations [Bay-0 × Shadahara (BaySha), Col-4 × Ler-0 (ColLer) and Cvi-0 × Ler-2 (CviLer)] were grown under a variety of environmental conditions (high vs. low Fe) and population sizes. Between 17 and 19 elements were measured for each population, with both macro- and microelements represented. We demonstrate that this variation is controlled by both Mendelian and quantitative trait loci, and that altering the environment has a large effect on which loci contribute to the observed differences.

## Results

### Variation in the shoot ionome of *Arabidopsis thaliana* accessions

The concentration of various elements in healthy *A. thaliana* shoot tissue from Col-0 varies over 4 orders of magnitude depending on the element and its biological function (Table 1). Macronutrients such as Mg, P, K and Ca accumulate more than 9,500 µg g^−1^ of the shoot dry weight, whereas micronutrients such as B, Mn, Fe, Co, Ni, Cu, Zn and Mo range in concentration from 1–100 µg g^−1^. Non-essential, potentially toxic trace elements such as As, Se, and Cd can accumulate to between 1–10 µg g^−1^ without any visible symptoms of toxicity. An ANOVA analysis of the accumulation in the 12 accessions reveals that variation in 13 of the 17 elements measured are under genetic control within the population (Table 1, Table 2, Table S1A). Several elements showed large variation between the lowest and highest accumulating accessions (Table 2). Mo showed the most variation with 44 significant pairwise differences between accessions (Table S1). Since Col-0 is the reference accession and a parent of many of the available RIL populations, we have also included a table of elemental differences compared with Col-0 (Table 2).

**Table 1:** Shoot and seed ionome of *A. thaliana* Col-0.

Element^1^	Shoot^2^			Seed^3^		
	*Average*	*SD*	*Line effect* 4	*Average*	*SD*	*Line effect*
Li	11.83	2.93	yes	0.87	0.51	no
B	43.88	9.75	yes	6.84	2.1	yes
Na	860.14	217.84	yes	64.82	14.63	yes
Mg	12900	2900	no	3283	614	yes
P	9700	900	yes	9937	1578	no
K	46100	4700	yes	10690	2319	no
Ca	45000	2900	yes	5645	551	no
Mn	63.59	12.94	yes	32.49	5.49	no
Fe	100.63	7.62	yes	42.52	18.57	yes
Co	1.86	0.16	yes	0.27	0.15	no
Ni	1.41	0.26	yes	0.36	0.14	yes
Cu	1.82	0.72	yes	1.67	1.14	no
Zn	61.06	14.24	yes	58.65	14.42	yes
As	1.04	1.95	no	1.34	0.48	no
Se	9.3	9.03	no	11.71	5.74	no
Mo	5.52	0.81	yes	1.01	0.41	yes
Cd	2.03	0.25	no	0.36	0.11	yes

1 All elements presented as µg g^−1^.

2 Data represents the average (n = 60 except for Li n = 30), individual plants harvested and analyzed in 3–6 separate experiments.

3 Data represents the average (n = 12) of individual samples from seed pooled from 4 plants sub sampled 3 times each and analyzed in 2 separate experiments.

4 Column indicates if the line effect is significant in the ANOVA.

**Table 2:** Shoot ionome variation across *A. thaliana* ecotypes compared to Col-0.

Name		Acc.#	B	Na	Mg	P	K	Ca	Mn	Fe	Co	Ni	Cu	Zn	As	Se	Mo	Cd
			*% difference from col-0*															
Cvi-0	Cape Verdi Islands	1096				23	27	−24	15	−24							46	−25
Est-1	Eastland, Russia	1150				17	37		24									
Kas-1	Kashmir, India	1264												−33			−59	−26
Mrk-0	Markt Baden, Germany	1374		−32			36		28		−22						−27	
Mt-0	Martuba Cyrenaika, Libya	1380				27	56		23								−28	
Se-0	San Eleno, Spain	1502						−14	19		73							
Ts-1	Tossa del Mar, Spain	1552		117			16				42							
Van-0	Vancouver, Canada	1584				25	53		45	12							−76	
Ws-0	Wassilewskija, Russia	1602	−31	89										−34			−81	−26
Nd-1	Niederzenz, Germany	1636					23		27								−19	
Tsu-1	Tsu, Japan	1640		127			20	21	23	21								
Ler-2		8581				−19			26	16							−73	

All element values are in percent difference from Col-0 with data representing the significant (student *t*-test P<0.01) average difference across 2 independent experiments (n = 10 individual plants per experiment).

### Variation in the seed ionome of *Arabidopsis thaliana* accessions

The concentration of elements measured in *A. thaliana* seed varies over 4 orders of magnitude, a similar scale to that observed in shoots (Table 1). However, there are several significant differences between the shoot and seed ionome, with certain elements being enriched or reduced relative to other elements. For example, on a µg g^−1^ dry weight basis, P does not change concentrations from seed to shoot, but K is approximately 4-fold lower in the seeds (Table 1). Furthermore, ANOVA analysis of the elemental composition of seeds from different *A. thaliana* accessions showed significant genetic control for 8 of the 17 elements (Table 1, Table 3, Table S1). Several significant differences between accessions in the seed mirror the differences observed between the accessions in the leaves, however, there are multiple comparisons in which significant differences in the leaves are not reflected in the seed (and vice versa). For example, the elevated shoot Na observed in Ts-1 is also reflected in the seed, with Ts-1 showing a 161% increase in seed Na compared to Col-0 (Table 2 and 3), while the other high shoot Na accession, Tsu-1 does not accumulate significantly different amounts of Na in its seeds compared to Col-0 (Table 3).

**Table 3:** Seed ionome variation across *A. thaliana* ecotypes compared to Col-0.

Name		Acc. #	Li	B	Na	Mg	P	K	Ca	Mn	Fe	Co	Ni	Cu	Zn	As	Se	Mo	Cd
			*% difference from col-0*																
Cvi-0	Cape Verdi Islands	1096							−23		27							69	
Est-1	Eastland, Russia	1150																	
Kas-1	Kashmir, India	1264	−58						−54		55						−83	−43	
Mrk-0	Markt Baden, Germany	1374																	
Mt-0	Martuba Cyrenaika, Libya	1380																	
Ee-0	San Eleno, Spain	1502											309		72				
Ts-1	Tossa del Mar, Spain	1552			161														
Van-0	Vancouver, Canada	1584	−58															−48	
Ws-0	Wassilewskija, Russia	1602																−58	−82
Nd-1	Niederzenz, Germany	1636																53	
Tsu-1	Tsu, Japan	1640				−12			−12									92	
Ler-2		8581		204	76			−34						69				−75	

All element values are in percent difference from Col-0 with data representing the significant (student *t*-test P<0.01) average difference across 2 independent experiments (n = 10 individual plants per experiment).

### Identification of QTLs controlling ionomic differences in three Recombinant Inbred Populations

To expand on the genetic characterization of the ionome, we analyzed the elemental composition of 3 RIL populations: Bay-0 × Shadahara (BaySha), Col-4 × Ler-0 (ColLer) and Cvi-0 × Ler-2 (CviLer). BaySha and CviLer were grown in two different environments (two growth media with differing ingredients) for a total of 5 different experiments. The experiments differed in the number of RIL lines analyzed (from 93 for ColLer to 411 for the second BaySha experiment) and the number of plants analyzed per line (1–3). The parents of the RIL population showed significant differences in 53 out of the possible 87 instances (17 elements×5 populations+S and Rb measured in the second BaySha experiment). We identified 218 QTLs in the five experiments although a significant number are likely the same QTL found in two experiments of the same population or are due to the shared Ler parent of CviLer and ColLer populations (Tables 4 and 5, Table S2). 158 of the 218 QTLs (72%) were found for elements for which the parents had a significant difference, for an average of 3.0 QTLs/element while elements in which the parents were not significantly different averaged 1.8 QTLs (Tables 4 and 5). All of the 19 major (r^2^>20%) QTLs we identified came from elements in which the parents were significantly different (Tables 4 and 5, Table S2). Frequency distributions showing difference between parental lines are provided in supplemental Files S1, S2, S3, S4, S5. To evaluate the effect of different experimental designs on the ability to identify QTLs for ionomic traits, we created subsets of the large BaySha experiment (411 lines at n = 2, ∼800 samples) and the large CviLer experiment (165 lines at n = 3). Randomly generated n = 1 and n = 2 subsets of the CviLer data identified 69% and 95% as many QTLs respectively as the n = 3 data from which they were derived. When data from the 165 lines used in the small BaySha experiment was extracted from the large BaySha 411 line experiment, 20% fewer QTLs were identified, suggesting that the different numbers of QTLs identified between the large and small BaySha experiments is due to the change in the number of lines.

**Table 4:** All QTLs for each element in the ColLer and BaySha RIL populations.

	ColLer (93 lines, n = 3)						BaySha Small (165 lines, n = 3)						BaySha Full (411 lines, n = 2)					
	Sunshine Soil, Low Fe						Sunshine Soil, High Fe						Promix Soil, High Fe					
	p-value	Herit-ability	HiloRIL sperc^a^	Trans-diff^b^	QTLs	Major^c^	p-value	Herit-ability	HiloRIL sperc^a^	Trans-diff^b^	QTLs	Major^c^	p-value	Hertit-ability	HiloRIL sperc^a^	Trans-diff^b^	QTLs	Major^c^
Li	1.8E-03	44%	16%	N	0	0	NA	46%	10%	N	2	0	NA	55%	17%	N	1	0
B	5.7E-09	57%	4%	N	1	0	NA	40%	18%	Y	3	0	4.2E-04	63%	24%	Y	3	0
Na	NA	51%	24%	N	0	0	5.5E-10	79%	47%	Y	2	1	4.9E-11	79%	42%	Y	7	1
Mg	6.1E-08	52%	16%	N	0	0	1.5E-12	62%	9%	Y	2	1	3.2E-15	70%	21%	Y	6	1
P	2.0E-14	63%	25%	N	1	0	2.9E-14	66%	10%	Y	7	0	3.1E-34	73%	13%	Y	9	0
S	NA	NA	NA	NA	NA	NA	NA	NA	NA	NA	NA	NA	1.8E-15	86%	38%	Y	8	2
K	NA	61%	21%	Y	2	0	1.8E-15	72%	17%	Y	8	1	1.7E-42	75%	15%	Y	7	1
Ca	NA	51%	16%	N	0	0	1.2E-15	59%	2%	Y	3	1	2.2E-29	72%	22%	Y	4	1
Mn	3.8E-18	53%	4%	N	0	0	NA	43%	15%	N	2	0	1.3E-10	60%	23%	Y	5	0
Fe	9.3E-11	50%	3%	N	1	0	NA	42%	7%	N	1	0	2.3E-07	66%	26%	Y	3	1
Co	NA	47%	19%	N	0	0	7.6E-11	47%	7%	N	1	0	NA	61%	34%	Y	4	0
Ni	NA	54%	15%	N	1	0	1.9E-04	39%	8%	N	0	0	3.0E-07	60%	13%	N	2	0
Cu	NA	38%	23%	N	0	0	NA	42%	10%	Y	5	0	4.4E-06	63%	19%	Y	3	0
Zn	2.7E-03	49%	14%	N	0	0	1.5E-03	47%	10%	Y	6	0	5.4E-09	57%	13%	Y	5	0
As	NA	98%	24%	NA	NA	NA	NA	55%	21%	N	0	0	NA	54%	18%	N	1	0
Se	NA	51%	29%	N	0	0	1.7E-03	55%	47%	Y	3	2	NA	57%	11%	Y	5	0
Rb	NA	NA	NA	NA	NA	NA	NA	NA	NA	NA	NA	NA	1.8E-35	71%	8%	Y	7	1
Mo	1.5E-16	66%	4%	N	2	0	5.0E-36	80%	2%	N	2	1	7.9E-49	80%	3%	N	2	1
Cd	NA	53%	20%	N	3	0	3.3E-06	46%	9%	N	1	0	2.7E-08	53%	10%	N	1	0

Population size and replicate number are included with each RIL as well as environment.

a Transgressive segregation as measured by the percentage of RILs significantly outside of the range of the parents.

b Transgressive segregation determined by the presence of QTLs with different directions of the additive effect.

c Major QTL with R^2^ value>0.20.

**Table 5:** All QTLs for each element in the CviLer RIL populations.

	CviLerLow (151 lines, n = 1)						CviLer High (161 lines, n = 3)					
	Sunshine Soil, Low Fe						Sunshine Soil, High Fe					
	p-value	Herit-ability	HiloRIL sperc^a^	Trans-diff^b^	QTLs	Major^c^	p-value	Herit-ability	HiloRIL sperc^a^	Trans-diff^b^	QTLs	Major^c^
Li	5.7E-05	NA	21%	N	1	0	2.2E-04	52%	34%	Y	2	1
B	NA	NA	27%	N	3	0	5.1E-16	70%	25%	Y	7	1
Na	1.4E-08	NA	13%	Y	1	1	2.7E-11	48%	7%	Y	4	0
Mg	3.3E-04	NA	24%	N	1	0	1.9E-31	59%	6%	Y	5	0
P	1.5E-05	NA	39%	N	3	0	1.4E-46	78%	2%	Y	5	1
S	NA	NA	NA	NA	NA	NA	NA	NA	NA	NA	NA	NA
K	NA	NA	25%	Y	2	0	7.0E-19	57%	12%	N	3	0
Ca	NA	NA	34%	N	1	0	1.0E-29	56%	6%	N	1	0
Mn	2.4E-07	NA	11%	N	3	0	NA	68%	40%	Y	6	0
Fe	NA	NA	28%	N	2	0	7.2E-05	51%	13%	N	2	0
Co	NA	NA	19%	N	1	0	NA	47%	12%	N	1	0
Ni	NA	NA	23%	N	0	0	2.2E-03	45%	24%	N	0	0
Cu	NA	NA	21%	N	1	0	NA	37%	15%	N	3	0
Zn	NA	NA	15%	N	3	0	NA	63%	41%	Y	5	0
As	NA	NA	26%	N	0	0	6.1E-11	54%	9%	N	0	0
Se	NA	NA	38%	N	2	0	NA	53%	13%	N	0	0
Rb	NA	NA	NA	NA	NA	NA	NA	NA	NA	NA	NA	NA
Mo	8.2E-08	NA	9%	N	3	1	1.0E-29	81%	4%	N	2	1
Cd	7.3E-05	NA	13%	N	1	0	1.2E-08	56%	11%	N	2	0

Population size and replicate number are included with each RIL as well as environment.

a Transgressive segregation as measured by the percentage of RILs significantly outside of the range of the parents.

b Transgressive segregation determined by the presence of QTLs with different directions of the additive effect.

c Major QTL with R^2^ value>0.20.

### Transgressive Segregation and Epistasis

We tested for transgressive segregation using two independent methods: the number of RILs which were significantly higher or lower than the parents grown in the same trays or having two QTLs with opposite allelic effects. For elements in which there was not a significant difference between the parents, the percentage of RILs that fell outside the range of the parents ranged from 10%–41% (Tables 4 and 5, Files S1, S2, S3, S4, S5). The likelihood of finding transgressive segregation by the opposite allelic effects test increased as the number of QTLs increased: transgressive segregation occurred in 14 of 19 elements in the large (411 line) BaySha experiment (83 QTLs), none of the elements in the ColLer experiment (11 QTLs) and only two of the elements in the first CviLer experiment (28 QTLs) (Tables 4 and 5). Epistatic interactions between the identified QTLs were examined using RQTL in the large BaySha population and the CviLer population experiments. Only five significant (p<0.01) interactions were found among the 53 (17+17+19) elements examined, none of which were found in the 411 line (large) BaySha population with the most power to detect epistatic interactions (Table S3).

### Environmental Effect on Element Correlations and QTL Discovery

**Figure 1:**
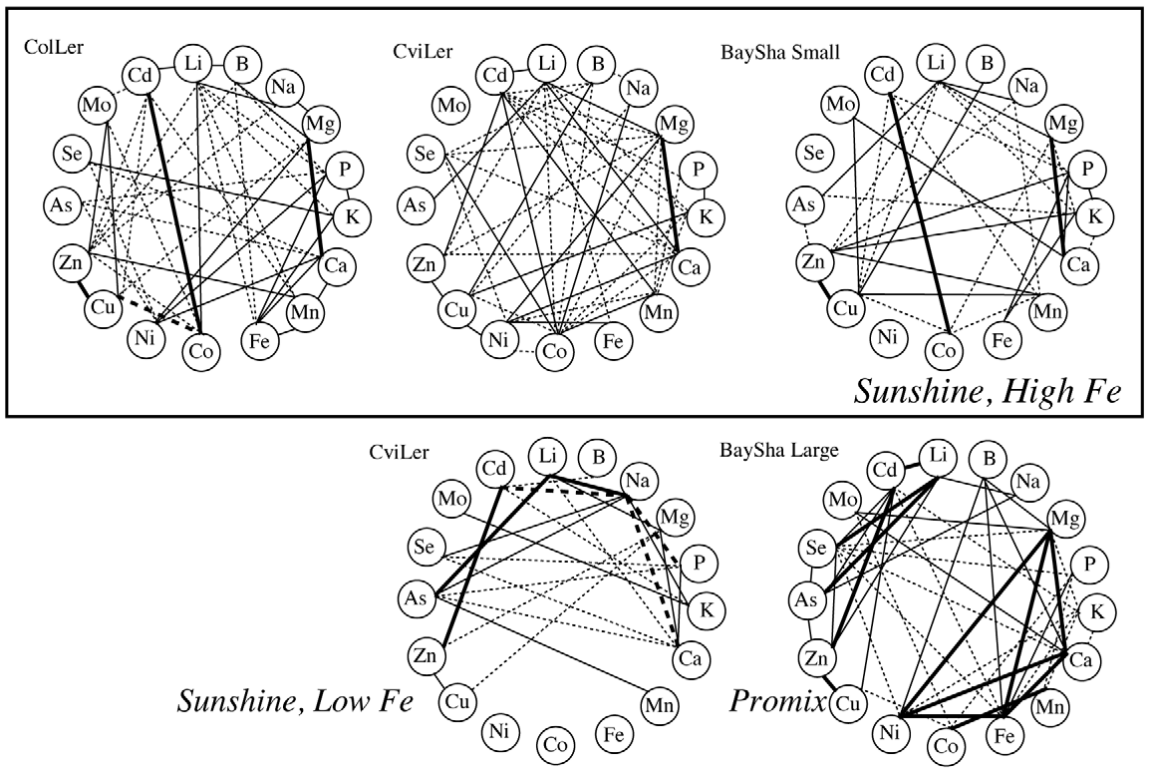
Elemental correlations for the 5 RIL populations. Solid lines represent a positive correlation value. Dashed lines represent negative correlation values. Thicker solid and dashed lines indicate correlations >0.5 or <−0.5, respectively. ColLer, CviLer high Fe, and BaySha small were all grown in Fe-sufficient Sunshine growth medium. The other CviLer population was grown in Sunshine growth medium with low Fe watering, while the Large BaySha population was grown in Promix growth medium.

Alterations in the environment or physiology of a plant can affect the accumulation of multiple elements simultaneously. Variation in mineral uptake in different environments has been described in *A. thaliana* [19], [21], [23] and *Silene vulgaris* [24]. The clearest example of the effect of the environment was observed in the relationships between elements within a given experiment. We measured the correlation between each pairwise combination of elements from every RIL in each of the five populations (Figure 1). While many elements were significantly correlated within each of the five experiments, only three pairs of elements were correlated in every population×environment we analyzed: Li-Na, Mg-Ca, and Cu-Zn, although Li-As, Li-Cd, Li-K, Li-Zn, P-Fe, Mg-Zn and Zn-Cd were found in 4 of the 5 experiments (Figure 1).

The BaySha and CviLer populations were each grown in two different growth media environments. Most of the significant elemental correlations (68 of 85 total correlations in BaySha and 66 of 76 total correlations in CviLer) were found in only one of the environments (Figure 1). The Ca-Mo correlation was unique to the BaySha population and was found in both environments. There is a three element correlated network that only appears in Sunshine growth medium with sufficient Fe, Co-Cd is positively correlated while both are negatively correlated to Cu (Cu-Cd is not significant in CviLer) (Figure 1). Interestingly, in the BaySha Promix experiment, Co-Cu is also negatively correlated, but Cu-Cd is positively correlated. Eight other correlations were shared between the three populations grown in sufficient Fe Sunshine growth medium. In the Fe sufficient Sunshine growth medium, BaySha shared 18 and 22 correlations with CviLer and ColLer, respectively (Figure 1). When the Promix grown BaySha population was compared with sufficient Fe Sunshine CviLer and ColLer populations, only 8 and 11 shared correlations were identified (Figure 1).

### Comparison of QTLs across environments

**Figure 2:**
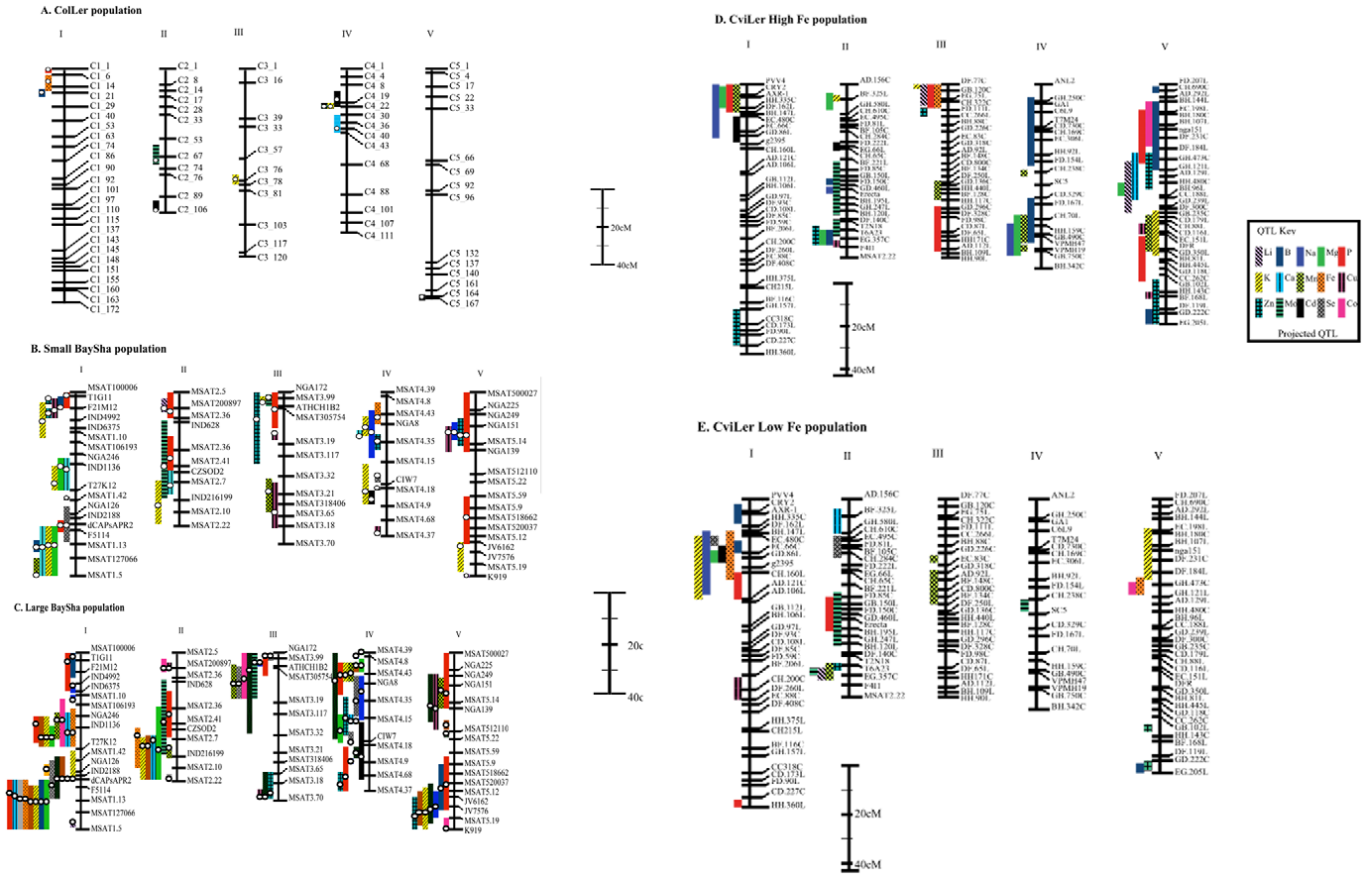
Chromosome maps with QTL noted for each element in which QTL were identified. The white circle within the colorful boxes represents the estimated location of the QTL. **A.** QTL identified in RIL ColLer. **B.** QTL identified in RIL BaySha, Sunshine growth medium. **C.** QTL identified in RIL BaySha, Promix growth medium. **D.** QTL identified in RIL CviLer, high Fe environment. **E.** QTL identified in RIL CviLer, low Fe environment.

To test whether altering the environment altered which genetic loci control the ionome, we compared the QTLs identified for each element in the five experiments. The only common QTL among the five populations, regardless of environment, population size or genetic background, is the Mo QTL on chromosome two corresponding to the MOT1 locus [25]. In the analysis of the parents grown with the CviLer population, seven of the elements measured, Li, Na, Mg, P, Ca, Mo and Cd, were significantly different in both growth conditions, three were not significantly different in either condition, and seven elements were only significant in one condition (Tables 4 and 5, Table S2). For the elements that were significantly different in the parents in both conditions, 4 of the 7 QTLs were found in both conditions (Tables 4 and 5, Figure 2, Table S2). In the analysis of the parents grown with the BaySha populations, nine of the elements measured, Na, Mg, P, K, Ca, Ni, Zn, Mo and Cd, were significantly different in both growing conditions, two were not significantly different in either condition, and eight elements were significantly different in only one condition (Tables 4 and 5, Table S2). Of the nine elements in both BaySha populations that were significantly different in the parents, 20 of 54 QTLs were found in both growing conditions (Tables 4 and 5, Figure 2, Table S2).

Comparing QTLs from the ColLer and CviLer populations grown in sufficient Fe Sunshine growth medium, we identified four common QTLs (Figure 2, Table S2). However, only one of those QTL (Mo) was shared with the BaySha population grown in the same medium. When comparing the BaySha populations with ColLer, three of the 11 ColLer QTLs appear to be in similar locations (Figure 2, Table S2). The CviLer populations grown in different environments share four common QTLs (Figure 2) and the BaySha populations share 25 QTLs (Figure 2, Table S2).

## Discussion

### Natural variation in the *A. thaliana* shoot and seed ionome

Identification of variation in elemental accumulation among *A. thaliana* accessions provides an excellent starting point for identifying genes important for regulation of the plant ionome, and for understanding how the ionome responds to different growth conditions and stresses. All of the *A. thaliana* accessions profiled in this study had at least one element that was significantly different (p<0.01) from the Col-0 reference accession. Several accessions, for example Est-1 and Nd-1, had no significant differences in elemental accumulation between them, even though they were collected from geographically distant sites [26]. One explanation is that they are adapted to soils with similar mineral contents. Unfortunately, we lack information on the type of soil from which most of these accessions were collected. The ionomic signature of Cvi is markedly different from that of all the other accessions in this study. The large difference in ionomic profiles between Cvi and the other accessions is mirrored in the genetic distance of Cvi from other accessions as shown by genome-scale analysis of sequence polymorphisms [26].

The variation observed in the accumulation of most elements between shoots and seeds may be attributed to the large differences in the biochemical and physiological functions of these tissues. For this reason, it is more appropriate to focus on element-to-element and accession-to-accession comparisons when contrasting the seed and shoot ionomes. Phosphorus is the fourth most abundant element in the shoots, but the second highest (and within 10% of K) in the seeds, likely reflecting the role of the P containing molecule phytate as the anion in storage crystals for cations like Ca, K, Mg, Mn and Zn [27]. Comparison of the seed Na accumulation of the three high shoot Na accessions suggests an active mechanism to either transport Na to the seed in Ts-1 or exclude Na from the seed in Ws-0 and Tsu-1. Of the 8 accessions that have shoot Mo levels that are significantly different from Col-0, 5 show similar differences in the seed ionome, while Nd-1 has lower Mo in the shoots, but higher Mo in the seeds and the remaining two lines are not significantly different in the seeds. The wide disparity in ionomic signatures of each accession in seeds and shoots suggests that the mechanisms governing elemental accumulation are distinct in these tissues. Waters and Grusak [28] demonstrated that both remobilization from the leaves and continued supply from the roots could contribute to seed mineral loading in Col-0, Cvi-0 and Ler-0, suggesting multiple control points in which natural variation could have different effects on the seed and leaf ionomes. Since conducting this screen, we have identified several of the genes underlying the variation detected in the leaves of the 12 accessions. We identified *HKT1*, encoding a Na-transporter, as the gene underlying the QTL responsible for elevated shoot Na in both Tsu-1 and Ts-1 [16], *MOT1*, a putative Mo transporter, as the gene underlying the low Mo in Ler-2, Ws-0 and Van-0 [17] and *FPN2*, an Fe and Co transporter as the gene responsible for increased Co in Ts-1 and Se-0 [18].

### Gene × Environment × Ionome Interactions

We detected a strong interaction between the environment and the genetic control of the ionome in our analysis of element-to-element genetic correlations and comparisons of QTLs detected in the same population in different environments. Two of the robust genetic correlations identified in all 5 experiments (Mg-Ca and Cu-Zn) have been identified by other researchers in a variety of species and environments [28]–[34]. The three robustly correlated pairs of elements (Li-Na, Mg-Ca and Cu-Zn) have similar chemical properties, suggesting that shared biochemical accumulation pathways accounts for the observed correlation. Many of the other significant correlations that we detected were specific to a given growth medium, RIL population or combination of both. This suggests that many of these relationships are not the result of a specific pathway for accumulating these elements, but are indirect effects of changes in the biochemistry or physiology of the plants in response to different environmental conditions such as the responses to low Fe and P identified by Baxter *et al.* [22]. In agreement with this hypothesis, we also observed a large number of environment-specific QTLs. For example in the CviLer population grown in high and low Fe conditions, the largest effect QTL we observed was for P in the Fe sufficient conditions, which explained 33% of the variance (Table S2). Smaller effect QTLs for Li, K and Fe were observed at the same location. Interestingly, none of the QTLs were observed when the RIL population was grown under low Fe conditions, which corresponds with the loss of any difference between Cvi-1 and L*er*-2 parent in P, K and Fe, and the correlations between Li-Fe, Li-K and P-K under low Fe. It has previously been observed that P status regulates Fe status, with high P reducing Fe accumulation and increasing expression of *IRT1* encoding the primary root Fe-transporter [35], [36]. It is possible that these responses are attributed to reduced Fe bioavailability in the growth medium caused by elevated P driving the precipitation of Fe as Fe-phosphate [37]. We also observed a set of co-localized QTLs on chromosome one and corresponding significant element correlations in the BaySha population: Mg, K and Ca had QTLs in both environments, while the Fe QTL and Mg-Fe and Ca-Fe correlations, were only found in the large BaySha, Promix growth medium experiment. Ghandilyan et al. [19] reported similar phenomena, observing that QTLs identified for multiple elements across populations were contingent on environment and type of organ (ie seed or tissue) the trait was measured, while Waters and Grusak [20] observed that a large number of QTLs for seed elemental accumulation in the ColLer population were not detected in all experiments conducted over a period of years.

### Implications of Experiment design and Parent Differences for QTL Discovery

As the QTL studies reported here required a considerable amount of effort and resources, we investigated several different experimental designs to optimize QTL discovery while limiting the number of samples analyzed. While we did detect some transgressive segregation, finding RIL populations in which the parents are different for the element of interest is clearly the best way to identify QTLs controlling a specific element. Across all experiments, major QTL(s), which are much easier to fine map, were far more likely to be found when the parents were significantly different for that element. The Ionomicshub database (www.ionomicshub.org) now has data for >350 accessions, including most available RIL population parents and is an excellent resource for finding accession pairs which differ for a given element. Reducing the number of replicates from 3 to 2 in the CviLer population (138 lines which had data for 3 samples) only reduced the number of QTLs identified by 5% while decreasing the number of lines in the large BaySha population from 411 to 165 reduced the number of QTLs identified by 22%. This suggests that for ionomics, like other traits [38], more lines are more beneficial than more replicates. With the possibility of analytical or biological outliers, we believe that n = 2 should be the minimum number of replicates per line. However, there does not appear to be a need to increase the number of plants analyzed for each line beyond two if it would reduce the number of lines analyzed or make the experiments cost or scope unfeasible.

Several studies examining elemental accumulation [19], [21], [23] in *A. thaliana* RIL populations have identified multiple epistatic interactions. In contrast, we found no significant (p-value<0.01) epistatic effects in the large BaySha population and only five significant epistatic effects in the two CviLer experiments, a few more than would be expected by chance alone. No epistatic interactions were found between QTLs identified using composite interval mapping (CIM). With 411 lines in the BaySha population we had more than sufficient statistical power to detect 2 way epistatic interactions. The difference among previously published studies and ours may be due to a more conservative permutation based significance cutoff in our Rqtl analysis. Ultimately, resolution of this question will require the cloning of genes underlying these QTLs and identifying epistatic interactions between the genes.

### Conclusion

We have demonstrated that natural accessions of *A. thaliana* provide an excellent resource for ionomic gene discovery. There is a strong effect of the growth environment on both the element-to-element correlations and the QTLs underlying elemental accumulation. All the ionomic data for shoot tissue discussed is publicly accessible for viewing, download and re-analysis at the online Purdue Ionomics Information Management System (PiiMS; accessed at www.ionomicshub.org).

## Materials and Methods

### Plant Growth

All of the seeds for the *A. thaliana* accessions used in this study were obtained from the ABRC. The accessions were planted in seven (5.25″×5.25″) pots or in two rows of a 20-row (10.5″×21″) tray. The planting pattern was varied across trays to reduce positional effects. Plants were grown in a climate-controlled room at 19–24°C with 10 hours of light at 80 to 100 µE for 36 to 40 days. The growth medium were Sunshine mix LB2 (Sun Gro Horticulture) (screened through a 1/4 inch mesh) and Promix (Premier Horticulture). Both mixtures are peat based, but they differ in the identity and grade of the other components. Notably, Sunshine has gypsum, while Promix has vermiculite as well as added macro- and micronutrients. Both growth media were amended with Li, Na, Co, Ni, As, Se, Rb, Sr and Cd at sub-toxic concentrations [39]. The CviLer low Fe (n = 1, 151 individuals) population was grown in Sunshine mix LB2 and watered with 0.25X Hoaglands solution+2.5ml/LFe tartrate. The CviLer (n = 3, 161 individuals) and BaySha small (n = 3, 165 individuals) population was grown in Sunshine mix LB2 and watered with 0.25X Hoaglands solution (File S7) with additional Fe (1ml Fe HBED/L). The large BaySha populations (n = 2, 411 individuals) was grown in Promix (Premier Horticulture) and also watered with 0.25X Hoaglands solution +1ml Fe HBED/L. ColLer population (n = 3, 93 individuals) was grown in Sunshine mix LB2 and watered with 0.25X Hoaglands solution+1ml Fe HBED/L. All plants were watered at 3 to 4 day intervals.

### Ionomic Analysis

Plants were sampled by removing 2–3 leaves (0.001–0.005 g fresh weight) and washing with 18 MΩ water before being placed in Pyrex digestion tubes. Sampled plant material was dried for 24 hr at 88°C, and weighed before open-air digestion in Pyrex tubes using 0.7 mL concentrated HNO_3_ (Mallinckrodt AR select grade) at 110°C for 5 hours. Each sample was diluted to 6.0 mL with 18 MΩ water and analyzed for Li, B, Na, Mg, P, K, Ca, Mn, Fe, Co, Ni, Cu, Zn, As, Se, Mo and Cd (and Rb and Sr in some experiments) on an Elan DRCe ICP-MS (PerkinElmer Sciex). NIST traceable calibration standards (ULTRAScientific, North Kingstown RI) were used for the calibration. Seed from two plants of each accession was obtained by increasing their day length to 24 hours. Three accessions (1372, 1602 and 1264) were kept at 4°C for 1 month to induce flowering. The seed was analyzed similarly to the plant tissue. The entire growth and analysis procedure was repeated to measure reproducibility. All experiments were managed using the Purdue Ionomics Information Management System (PiiMS) [40], and all ionomic data is publicly available for viewing, download and reanalysis at www.ionomicshub.org.

### Correlation analysis

For each pairwise combination of elements in the experiment, Pearson correlation coefficients were found using the tray centered sample data for pairwise complete observations utilizing the corr function in R. Statistically significant correlations were identified using the t-distribution with n-2 degrees of freedom (where n = 411 in the BaySha data) where t = (corr * sqrt(n−2))/(sqrt(1−corr^2^)), or equivalently using the F-distribution with 1 and n-2 degrees of freedom where F = (corr^2^ * (n−2))/(1−corr^2^). A conservative Bonferroni correction was applied to the alpha level of 0.05 to adjust for the 19 elements. A total of 171 pairwise elemental combinations exist (19 choose 2 = (19)*(19−1)/2 = 171). Thus, only correlations having a p-value below 0.05/171 (∼2.924×10^−4^) were identified as being significant.

### QTL Analysis

Each tray for each of the five populations examined was normalized as follows. The data for each element was divided into quartiles and the Inter Quartile Range (IQR), the upper and lower bounds of the middle two quartiles, was determined. Element concentration, which was outside the range of lower bound minus 3 times IQR to upper bound plus 3 times IQR, was removed. Each tray was centered so that the average of the two parent lines grown in the tray was the same across all trays. The mean value across all trays for each line was then used for QTL analysis. The marker sets were obtained from the Natural website (CviLer, www.dpw.wau.nl/natural/), the BaySha website (http://dbsgap.versailles.inra.fr/vnat/Documentation/33/DOC.html) and Singer *et al.* [41] for ColLer. We used reduced marker sets for the CviLer and ColLer mapping. The marker maps for all QTL mapping experiments are included as supplemental File S6. Note that the chromosome numbering and orientation does not match the final QTL results, as we changed the output values to match the published maps. We performed composite interval mapping (CIM) using QTL Cartographer version 1.17f [42], with CIM [43], [44] model 6, a walk speed of 2cM, a window of 5 cM, using the forward and backward regression method. To determine threshold values, the permutation method was used [45] with 1000 permutations per element per population (Table S3).

After locating all main effect (single) QTL, epistatic interactions between two loci were investigated using the scantwo function in the software R/qtl [46]. Tests were conducted between all pairwise loci (both within and between chromosomes) using the Haley-Knott regression with a 2 cM walking speed. One thousand permutations were performed for each of the ionomic traits to determine the genome-wide significance threshold.

### Experimental Design

To test the effects of changing the number of replicates or the number of lines, we performed two in silico experiments using the QTL data. 1. From the CviLer high Fe population, we took the 138 lines in which 3 samples were analyzed per line and made 20 subsets of the outlier removed, tray centered, data: 10 subsets in which 2 samples were randomly selected from each line and 10 subsets in which 1 sample was selected from each line. We then performed QTL analysis as described above on the 20 subsets as well as the full n = 3 data for the 138 lines. Table S4a contains the mean number of QTLs identified within each set of 10 experiments. 2. We took the data from the large BaySha population and made a subset of the 165 lines that were analyzed in the small BaySha population performed QTL analysis as described above. A comparison of the number of QTLs identified in this experiment with that of the full 411 lines is shown in Table S4b.

## Supporting Information

Table S1 Significant pairwise comparisons between 12 accessions of A. thaliana. Line effect indicates significant differences (p<0.05) between accessions for that element. A. A. thaliana leaf data. B. A. thaliana seed data.
(0.03 MB DOC)

Table S2 All QTL for each element across all 5 RIL populations. LOD (logarithm of the odds) score above the LOD threshold is indicated for each QTL. QTL region are indicated by MI start and MI end and the projected QTL location is given in cM. Cofactors indicates the number of cofactors used in the CIM model. Threshold values indicate the 99% confidence interval derived from 1000 permutations.
(0.37 MB DOC)

Table S3 Two-way epistatic interactions for each RIL population across all 5 chromosomes. Lod.full is the log-odds ratio of the full model with two loci and their interaction compared to the null model with no QTL. Lod.fv1 is the log-odds ratio of the full model compared to the best single QTL model with one locus on either chromosome A or B (not necessarily at the same location as the full model loci). Lod.int is the log-odds ratio of the interaction term which is found by comparing the full model with an interaction term, to the two QTL model with no interaction term. Lod.add is the log-odds ratio of the additive effects, found by comparing the two QTL model (no interaction term) to the null model with no QTL. Lod.av1 is the log-odds ratio comparing the two QTL model with no interaction term, to the best single QTL model with one locus on either chromosome A or B (not necessarily at the same location as in the two QTL model).
(0.04 MB DOC)

Table S4 Results of in silico experimental design simulations. A. Number of QTLs detected in the subset of CviLer lines with 3 samples analyzed. First two rows indicate the average of 10 randomly generated subsets with n = 1 or 2, third row indicates the number of QTLs identified with the full n = 3 dataset. B. QTLs identified from the large BaySha experiment when either the full 411 lines or the subset of 165 lines corresponding to the smaller BaySha set was used.
(0.05 MB DOC)

File S1 Frequency plots of parental lines and RILs for each element across the 5 RIL populations. X-axis represents the centered PPM (See Methods) of indicated element. Y-axis indicates frequency of occurrence. Black vertical lines indicate the 95% confidence interval of the parents distribution (i.e. lower parent−1.96 SD (pooled) to higher parent+1.96 SD(pooled)) 1.1. Comparison of ColLer.
(0.06 MB PDF)

File S2 Frequency plots of parental lines and RILs for each element across the 5 RIL populations. X-axis represents the centered PPM (See Methods) of indicated element. Y-axis indicates frequency of occurrence. Black vertical lines indicate the 95% confidence interval of the parents distribution (i.e. lower parent−1.96 SD (pooled) to higher parent+1.96 SD(pooled)) 2. Comparison of BaySha, grown in Sunshine Soil.
(0.06 MB PDF)

File S3 Frequency plots of parental lines and RILs for each element across the 5 RIL populations. X-axis represents the centered PPM (See Methods) of indicated element. Y-axis indicates frequency of occurrence. Black vertical lines indicate the 95% confidence interval of the parents distribution (i.e. lower parent−1.96 SD (pooled) to higher parent+1.96 SD(pooled)). Comparison BaySha, grown in Promix soil.
(0.06 MB PDF)

File S4 Frequency plots of parental lines and RILs for each element across the 5 RIL populations. X-axis represents the centered PPM (See Methods) of indicated element. Y-axis indicates frequency of occurrence. Black vertical lines indicate the 95% confidence interval of the parents distribution (i.e. lower parent−1.96 SD (pooled) to higher parent+1.96 SD(pooled)). Comparison of CviLer, high Fe environment.
(0.06 MB PDF)

File S5 Frequency plots of parental lines and RILs for each element across the 5 RIL populations. X-axis represents the centered PPM (See Methods) of indicated element. Y-axis indicates frequency of occurrence. Black vertical lines indicate the 95% confidence interval of the parents distribution (i.e. lower parent−1.96 SD (pooled) to higher parent+1.96 SD(pooled)). Comparison of CviLer, low Fe environment.
(0.06 MB PDF)

File S6 Estimated genetic maps. Estimated genetic maps using marker data for our RIL populations in QTL Cartographer display. Map function and unit of measurement are included at the top of each map estimate followed by number of chromosomes, total number of markers mapped, mean and standard deviation for markers and inter-marker distance. The table is a representation of marker distance between markers, across all 5 Arabidopsis thaliana chromosomes. Finally, a list of marker name and order across chromosomes is included for each population. 1. Map data for the BaySha populations. 2. Map data for the ColLer population. 3. Map data for the CviLer Low Fe population. 4. Map data for the CviLer High Fe population.
(0.03 MB TXT)

File S7 Hoaglands Media Recipe. Modified Hoaglands media used in this study.
(0.04 MB DOC)
