# Supplementary material for: Natural Genetic Variation in Selected Populations of Arabidopsis thaliana Is Associated with Ionomic Differences
Source: PLoS One. 2010 Jun 14;5(6):e11081. doi: 10.1371/journal.pone.0011081 (PMC2885407; doi:10.1371/journal.pone.0011081)
Supplement: Table S1 — Significant pairwise comparisons between 12 accessions of A. thaliana. Line effect indicates significant differences (p<0.05) between accessions for that element. A. A. thaliana leaf data. B. A. thaliana seed data. (0.03 MB DOC) [file pone.0011081.s001.doc]

**Supplemental Table 1**

**A. Result summary (data=cleaned Leaf) – (9/18/2007)**

| Element | Line effect | Significant pairwise difference (leaf) |
| --- | --- | --- |
| Li | Yes | [Col-0 Cvi-0]; [Col-0 Ler-2]; [Cvi-0 Ler-2]; [Cvi-0 Tsu-1]; [Cvi-0 Van-0]; [Ler-2 Nd-1]; [Ler-2 Ts-1] |
| B | Yes | [Col-0 Cvi-0]; [Cvi-0 Kas-1]; [Cvi-0 Mt-0]; [Cvi-0 Nd-1]; [Cvi-0 Se-0]; [Cvi-0 Ws-0]; [Est-1 Ws-0]; [Ler-2 Ws-0]; [Mrk-0 Ws-0] |
| Na | Yes | [Col-0 Ts-1]; [Col-0 Tsu-1]; [Col-0 Ws-0]; [Cvi-0 Ts-1]; [Cvi-0 Tsu-1]; [Cvi-0 Ws-0]; [Est-1 Ts-1]; [Est-1 Tsu-1]; [Est-1 Ws-0]; [Kas-1 Ts-1]; [Kas-1 Ws-0]; [Ler-2 Ts-1]; [Ler-2 Tsu-1]; [Ler-2 Ws-0]; [Mrk-0 Ts-1]; [Mrk-0 Tsu-1]; [Mrk-0 Ws-0]; [Mt-0 Ts-1]; [Mt-0 Tsu-1]; [Mt-0 Ws-0]; [Nd-1 Ts-1]; [Nd-1 Tsu-1]; [Nd-1 Ws-0]; [Se-0 Ts-1]; [Se-0 Tsu-1]; [Se-0 Ws-0]; [Ts-1 Van-0]; [Tsu-1 Van-0]; [Van-0 Ws-0] |
| Mg | No | none |
| P | Yes | [Col-0 Cvi-0]; [Col-0 Est-1]; [Col-0 Ler-2]; [Col-0 Mt-0]; [Col-0 Van-0]; [Cvi-0 Ler2]; [Cvi-0 Se-0]; [Cvi-0 Ts-1]; [Cvi-0 Tsu-1]; [Cvi-0 Ws-0]; [Est-1 Ler2]; [Est-1 Tsu-1]; [Kas1 Ler2]; [Ler-2 Mrk-0]; [Ler-2 Mt-0]; [Ler-2 Nd-1]; [Ler-2 Ts-1]; [Ler-2 Van-0]; [Mrk-0 Tsu-1]; [Mt-0 Se-0]; [Mt-0 Ts-1]; [Mt-0 Tsu-1]; [Mt-0 Ws-0]; [Ts-1 Van-0]; [Tsu-1 Van-0]; [Van-0 Ws-0] |
| K | Yes | [Col-0 Cvi-0]; [Col-0 Est-1]; [Col-0 Mrk-0]; [Col-0 Mt-0]; [col-0 Van-0]; [Ler-2 Mt-0]; [Ler-2 Van-0]; [Mt-0 Se-0]; [Mt-0 Ts-1]; [Mt-0 Tsu-1]; [Mt-0 Ws-0]; [Tsu-1 Van-0]; [Van-0 Ws-0] |
| Ca | Yes | [Cvi-0 Ler-2]; [Cvi-0 Mt-0]; [Cvi-0 Tsu-1]; [Cvi-0 Van-0] |
| Mn | Yes | [Col-0 Van-0]; [Ts-1 Van-0] |
| Fe | Yes | [Cvi-0 Ler-2]; [Cvi-0 Van-0] |
| Co | Yes | [Col-0 Mrk-0]; [Col-0 Se-0]; [Col-0 Ts-1]; [Cvi-0 Mrk-0]; [Cvi-0 Se-0]; [Cvi-0 Ts-1]; [Est-1 Se-0]; [Est-1 Ts-1]; [Kas-1 Se-0]; [Kas-1 Ts-1]; [Ler-2 Mrk-0]; [Ler-2 Se-0]; [Ler-2 Ts-1]; [Ler-2 Tsu-1]; [Mrk-0 Mt-0]; [Mrk-0 Se-0]; [Mrk-0 Ts-0]; [Mrk-0 Van-0]; [Mt-0 Se-0]; [Mt-0 Ts-1]; [Mt-0 Tsu-1]; [Nd-1 Se-0]; [Nd-1 Ts-1]; [Se-0 Tsu-1]; [Se-0 Van-0]; [Se-0 Ws-0]; [Ts-1 Tsu-1]; [Ts-1 Van-0]; [Ts-1 Ws-0] |
| Ni | Yes | [Col-0 Ler-2]; [Cvi-0 Ler-2]; [Cvi-0 Mt-0] |
| Cu | Yes | none |
| Zn | Yes | none |
| As | No | none |
| Se | No | none |
| Mo | Yes | [Col-0 Cvi-0]; [Col-0 Kas-1]; [Col-0 Ler-2]; [Col-0 Van-0]; [Col-0 Ws-0]; [Cvi-0 Est-1]; [Cvi-0 Kas-1]; [Cvi-0 Ler-2]; [Cvi-0 Mrk-0]; [Cvi-0 Mt-0]; [Cvi-0 Nd-1]; [Cvi-0 Ts-1]; [Cvi-0 Tsu-1]; [Cvi-0 Van-0]; [Cvi-0 Ws-0]; [Est-1 Ler-2]; [Est-1 Van-0]; [Est-1 Ws-0]; [Kas-1 Se-0]; [Kas-1 Ts-1]; [Kas-1 Tsu-1]; [Ler-2 Mrk-0]; [Ler-2 Mt-0]; [Ler-2 Nd-1]; [Ler-2 Se-0]; [Ler-2 Ts-1]; [Ler-2 Tsu-1]; [Mrk-0 Van-0]; [Mrk-0 Ws-0]; [Mt-0 Van-0]; [Mt-0 Ws-0]; [Nd-1 Van-0]; [Nd-1 Ws-0]; [Se-0 Van-0]; [Se-0 Ws-0]; [Ts-1 Van-0]; [Ts-1 Ws-0]; [Tsu-1 Van-0]; [Tsu-1 Ws-0] |
| Cd | No | none |

B. Result summary (Data = clean seed)

| Element | Line effect | Significant pairwise difference (seed) |
| --- | --- | --- |
| Li | No | none |
| B | Yes | none |
| Na | Yes | [Est1-Ts1]; [Kas1-Ts1] |
| Mg | Yes | [Col1-Tsu1]; [Col0-Van0] |
| P | No | none |
| K | No | none |
| Ca | No | none |
| Mn | No | none |
| Fe | Yes | [Mrk0-Se0] |
| Co | No | none |
| Ni | Yes | [Kas1-Se0] |
| Cu | No | none |
| Zn | Yes | [Est1-Mrk0]; [Est1-Se0]; [Est1-Ts1]; [Est1-Tsu1]; [Est1-Van0] |
| As | No | none |
| Se | No | none |
| Mo | Yes | [Col0-Ler2]; [Cvi0-Kas1]; [Cvi0-Ler2]; [Ler2-Mrk0]; [Ler2-Mt0]; [Ler2-Nd1]; [Ler2-Tsu1]; [Ler2-Van0] |
| Cd | Yes | [Cvi0-Ler2]; [Est1-Ler2] |
