# Supplementary material for: Natural Genetic Variation in Selected Populations of Arabidopsis thaliana Is Associated with Ionomic Differences
Source: PLoS One. 2010 Jun 14;5(6):e11081. doi: 10.1371/journal.pone.0011081 (PMC2885407; doi:10.1371/journal.pone.0011081)
Supplement: Table S2 — All QTL for each element across all 5 RIL populations. LOD (logarithm of the odds) score above the LOD threshold is indicated for each QTL. QTL region are indicated by MI start and MI end and the projected QTL location is given in cM. Cofactors indicates the number of cofactors used in the CIM model. Threshold values indicate the 99% confidence interval derived from 1000 permutations. (0.37 MB DOC) [file pone.0011081.s002.doc]

| Population | Element | Chrm | QTL location (cM) | LOD score | MI Start | MI End | r^2^ | Additive effect | Closest marker | Total variation accounted for by all QTLs for a trait | Cofactors | Threshold LR value | Threshold LOD value |
| --- | --- | --- | --- | --- | --- | --- | --- | --- | --- | --- | --- | --- | --- |
| CviLerHighFe | As | --- | --- | --- | --- | --- | --- | --- | --- | --- | 6 | 11.99 | 2.60 |
| CviLerHighFe | B | 2 | 72.21 | 3.73 | 68.21 | 74.21 | 0.05 | -3.26 | EC.235LCol247C | 0.58 | 10 | 11.77 | 2.56 |
| CviLerHighFe | B | 2 | 46.72 | 2.69 | 46.72 | 46.72 | 0.03 | 2.44 | FD.150C | 0.58 | 10 | 11.77 | 2.56 |
| CviLerHighFe | B | 4 | 24.71 | 5.30 | 7.47 | 38.05 | 0.07 | 3.42 | EC.306L | 0.58 | 10 | 11.77 | 2.56 |
| CviLerHighFe | B | 4 | 61.66 | 15.33 | 54.37 | 73.86 | 0.21 | -6.30 | HH.159CCol | 0.58 | 10 | 11.77 | 2.56 |
| CviLerHighFe | B | 5 | 3.46 | 2.83 | 3.46 | 3.46 | 0.03 | 2.47 | CH.690C | 0.58 | 10 | 11.77 | 2.56 |
| CviLerHighFe | B | 5 | 19.27 | 4.80 | 9.27 | 39.78 | 0.07 | 3.63 | AD.114CCol | 0.58 | 10 | 11.77 | 2.56 |
| CviLerHighFe | B | 5 | 117.03 | 8.64 | 105.79 | 117.03 | 0.11 | -4.21 | HH.122C120L | 0.58 | 10 | 11.77 | 2.56 |
| CviLerHighFe | Ca | 5 | 47.14 | 6.32 | 33.11 | 54.02 | 0.15 | 1345.47 | AD.129LCol | 0.15 | 5 | 12.02 | 2.61 |
| CviLerHighFe | Cd | 1 | 16.64 | 4.38 | 14.64 | 26.16 | 0.09 | -0.24 | EC.480C | 0.16 | 5 | 11.43 | 2.48 |
| CviLerHighFe | Cd | 2 | 28.30 | 2.91 | 28.30 | 30.90 | 0.06 | -0.19 | FD.222LCol | 0.16 | 5 | 11.43 | 2.48 |
| CviLerHighFe | Co | 5 | 17.27 | 3.19 | 9.27 | 28.74 | 0.12 | -0.10 | AD.114CCol | 0.12 | 2 | 10.98 | 2.38 |
| CviLerHighFe | Cu | 5 | 97.87 | 2.54 | 97.87 | 99.87 | 0.06 | 0.13 | GB.102LCol105C | 0.18 | 8 | 11.64 | 2.53 |
| CviLerHighFe | Cu | 5 | 67.75 | 2.93 | 65.89 | 69.98 | 0.07 | -0.15 | CD.179L | 0.18 | 8 | 11.64 | 2.53 |
| CviLerHighFe | Cu | 2 | 74.21 | 2.59 | 74.21 | 74.21 | 0.06 | -0.12 | EC.235LCol247C | 0.18 | 8 | 11.64 | 2.53 |
| CviLerHighFe | Fe | 3 | 0.01 | 6.67 | 0.01 | 10.73 | 0.14 | 3.68 | DF.77C | 0.21 | 8 | 11.83 | 2.57 |
| CviLerHighFe | K | 3 | 2.01 | 3.35 | 0.01 | 2.01 | 0.07 | 2385.09 | GB.120CColGAPC | 0.26 | 8 | 11.93 | 2.59 |
| CviLerHighFe | K | 5 | 65.89 | 6.54 | 59.60 | 79.98 | 0.14 | -3136.61 | CD.179L | 0.26 | 8 | 11.93 | 2.59 |
| CviLerHighFe | K | 2 | 7.23 | 2.62 | 7.23 | 7.23 | 0.05 | 2126.36 | BF.325L | 0.26 | 8 | 11.93 | 2.59 |
| CviLerHighFe | Li | 3 | 0.01 | 6.10 | 0.01 | 8.73 | 0.11 | -0.39 | DF.77C | 0.32 | 6 | 11.79 | 2.56 |
| CviLerHighFe | Li | 5 | 49.14 | 10.51 | 37.78 | 59.60 | 0.20 | 0.52 | HH.480C | 0.32 | 6 | 11.79 | 2.56 |
| CviLerHighFe | Mg | 1 | 6.07 | 3.15 | 2.07 | 10.80 | 0.07 | 398.65 | AXR1 | 0.38 | 7 | 11.83 | 2.57 |
| CviLerHighFe | Mg | 2 | 8.47 | 3.06 | 5.23 | 12.47 | 0.06 | 367.86 | BF.325L | 0.38 | 7 | 11.83 | 2.57 |
| CviLerHighFe | Mg | 2 | 72.21 | 3.09 | 68.21 | 74.21 | 0.07 | 398.46 | EC.235LCol247C | 0.38 | 7 | 11.83 | 2.57 |
| CviLerHighFe | Mg | 4 | 76.48 | 6.04 | 61.66 | 80.48 | 0.13 | 537.05 | GB.750C | 0.38 | 7 | 11.83 | 2.57 |
| CviLerHighFe | Mg | 5 | 49.14 | 2.98 | 47.14 | 52.02 | 0.06 | 361.69 | HH.480C | 0.38 | 7 | 11.83 | 2.57 |
| CviLerHighFe | Mn | 1 | 2.07 | 8.68 | 0.07 | 12.80 | 0.17 | -11.82 | PVV4 | 0.52 | 10 | 11.19 | 2.43 |
| CviLerHighFe | Mn | 3 | 49.20 | 3.76 | 44.50 | 53.20 | 0.06 | 7.96 | HH.440L | 0.52 | 10 | 11.19 | 2.43 |
| CviLerHighFe | Mn | 4 | 43.47 | 2.96 | 41.47 | 43.47 | 0.05 | 7.68 | g4539 | 0.52 | 10 | 11.19 | 2.43 |
| CviLerHighFe | Mn | 4 | 67.86 | 7.06 | 61.66 | 73.86 | 0.13 | -11.38 | GB.490C | 0.52 | 10 | 11.19 | 2.43 |
| CviLerHighFe | Mn | 4 | 76.48 | 2.60 | 76.48 | 78.48 | 0.05 | -7.39 | GB.750C | 0.52 | 10 | 11.19 | 2.43 |
| CviLerHighFe | Mn | 5 | 67.75 | 3.53 | 61.60 | 77.98 | 0.06 | 7.33 | CD.179L | 0.52 | 10 | 11.19 | 2.43 |
| CviLerHighFe | Mo | 2 | 51.98 | 37.17 | 37.30 | 60.61 | 0.73 | -0.22 | ErectaGPA1 | 0.78 | 5 | 11.51 | 2.50 |
| CviLerHighFe | Mo | 2 | 68.21 | 3.29 | 62.87 | 70.21 | 0.04 | -0.06 | DF.140C | 0.78 | 5 | 11.51 | 2.50 |
| CviLerHighFe | Na | 1 | 14.64 | 7.21 | 0.07 | 24.18 | 0.16 | 93.18 | HH.335CColPhyA | 0.41 | 8 | 11.39 | 2.47 |
| CviLerHighFe | Na | 2 | 50.52 | 2.49 | 50.52 | 50.52 | 0.04 | 66.71 | ErectaGPA1 | 0.41 | 8 | 11.39 | 2.47 |
| CviLerHighFe | Na | 4 | 69.86 | 5.58 | 65.86 | 80.48 | 0.12 | 81.59 | GB.490C | 0.41 | 8 | 11.39 | 2.47 |
| CviLerHighFe | Na | 5 | 19.27 | 3.64 | 11.27 | 24.74 | 0.09 | -72.34 | AD.114CCol | 0.41 | 8 | 11.39 | 2.47 |
| CviLerHighFe | Ni | --- | --- | --- | --- | --- | --- | --- | --- |  | 0 | 11.68 | 2.53 |
| CviLerHighFe | P | 1 | 4.07 | 10.36 | 0.07 | 12.80 | 0.11 | 515.79 | AXR1 | 0.72 | 10 | 11.46 | 2.49 |
| CviLerHighFe | P | 3 | 0.01 | 29.93 | 0.01 | 10.73 | 0.37 | -949.40 | DF.77C | 0.72 | 10 | 11.46 | 2.49 |
| CviLerHighFe | P | 3 | 65.57 | 6.38 | 56.31 | 75.76 | 0.06 | -382.52 | FD.98C | 0.72 | 10 | 11.46 | 2.49 |
| CviLerHighFe | P | 5 | 24.74 | 12.55 | 13.27 | 37.11 | 0.13 | -570.78 | AD.114CCol | 0.72 | 10 | 11.46 | 2.49 |
| CviLerHighFe | P | 5 | 81.98 | 5.44 | 71.98 | 92.17 | 0.05 | -344.05 | AD.75CCol | 0.72 | 10 | 11.46 | 2.49 |
| CviLerHighFe | Se | --- | --- | --- | --- | --- | --- | --- | --- |  | 4 | 11.62 | 2.56 |
| CviLerHighFe | Zn | 1 | 116.28 | 3.97 | 105.12 | 121.11 | 0.08 | 3.72 | CC.318C | 0.44 | 7 | 11.80 | 2.56 |
| CviLerHighFe | Zn | 2 | 72.21 | 4.43 | 66.21 | 74.21 | 0.10 | -4.24 | EC.235LCol247C | 0.44 | 7 | 11.80 | 2.56 |
| CviLerHighFe | Zn | 3 | 13.63 | 2.76 | 10.73 | 13.63 | 0.06 | 3.08 | FD.111LCol136C | 0.44 | 7 | 11.80 | 2.56 |
| CviLerHighFe | Zn | 5 | 37.78 | 4.64 | 33.11 | 49.14 | 0.09 | -3.88 | GH.117C | 0.44 | 7 | 11.80 | 2.56 |
| CviLerHighFe | Zn | 5 | 105.79 | 4.68 | 92.17 | 113.03 | 0.11 | 4.08 | BF.168LCol | 0.44 | 7 | 11.80 | 2.56 |
| CviLerLowFe | As | --- | --- | --- | --- | --- | --- | --- | --- |  | 2 | 11.84 | 2.57 |
| CviLerLowFe | B | 1 | 7.17 | 3.19 | 3.17 | 9.83 | 0.08 | 15.56 | AXR_1 | 0.27 | 4 | 11.18 | 2.43 |
| CviLerLowFe | B | 1 | 19.89 | 2.76 | 17.89 | 21.89 | 0.08 | 15.82 | EC.66C | 0.27 | 4 | 11.18 | 2.43 |
| CviLerLowFe | B | 5 | 113.92 | 3.59 | 108.59 | 117.92 | 0.10 | -17.38 | DF.119L | 0.27 | 4 | 11.18 | 2.43 |
| CviLerLowFe | Ca | 2 | 8.16 | 3.69 | 4.83 | 14.16 | 0.10 | 4042.99 | BF.325L | 0.10 | 6 | 12.21 | 2.65 |
| CviLerLowFe | Cd | 1 | 23.66 | 3.34 | 19.89 | 26.12 | 0.09 | -2.45 | GD.86L | 0.09 | 2 | 11.82 | 2.57 |
| CviLerLowFe | Co | 5 | 36.37 | 2.72 | 34.37 | 38.37 | 0.08 | -1.41 | GH.117C | 0.08 | 4 | 11.86 | 2.57 |
| CviLerLowFe | Cu | 1 | 76.02 | 3.08 | 74.02 | 82.04 | 0.08 | 1.75 | BF.206L_Col | 0.08 | 4 | 10.77 | 2.34 |
| CviLerLowFe | Fe | 1 | 19.89 | 6.21 | 13.53 | 33.16 | 0.15 | -16.15 | EC.66C | 0.26 | 7 | 12.39 | 2.69 |
| CviLerLowFe | Fe | 5 | 34.37 | 4.16 | 32.37 | 38.37 | 0.11 | -12.60 | DF.184L_Col | 0.26 | 7 | 12.39 | 2.69 |
| CviLerLowFe | K | 1 | 24.12 | 6.43 | 15.89 | 41.16 | 0.16 | 11990.83 | GD.86L | 0.27 | 6 | 12.17 | 2.64 |
| CviLerLowFe | K | 5 | 17.90 | 3.78 | 11.90 | 31.48 | 0.11 | -10209.30 | AD.114C_Col | 0.27 | 6 | 12.17 | 2.64 |
| CviLerLowFe | Li | 2 | 73.05 | 3.01 | 69.05 | 73.05 | 0.08 | 3.93 | EC.235L_Col_247C | 0.08 | 3 | 11.00 | 2.39 |
| CviLerLowFe | Mg | 1 | 23.66 | 3.14 | 21.89 | 26.12 | 0.07 | -1811.21 | GD.86L | 0.07 | 5 | 11.41 | 2.48 |
| CviLerLowFe | Mn | 3 | 23.71 | 2.68 | 23.71 | 25.71 | 0.06 | 78.14 | EC.83C_84L | 0.23 | 9 | 11.67 | 2.53 |
| CviLerLowFe | Mn | 3 | 37.62 | 2.94 | 30.06 | 43.32 | 0.06 | 75.37 | GB.210L | 0.23 | 9 | 11.67 | 2.53 |
| CviLerLowFe | Mn | 2 | 73.05 | 5.16 | 67.05 | 73.05 | 0.10 | 96.91 | EC.235L_Col_247C | 0.23 | 9 | 11.67 | 2.53 |
| CviLerLowFe | Mo | 4 | 43.95 | 2.93 | 41.95 | 46.14 | 0.05 | -0.17 | g4539 | 0.51 | 6 | 11.94 | 2.59 |
| CviLerLowFe | Mo | 2 | 71.05 | 2.77 | 69.05 | 71.05 | 0.06 | -0.20 | EC.235L_Col_247C | 0.51 | 6 | 11.94 | 2.59 |
| CviLerLowFe | Mo | 2 | 51.85 | 15.65 | 38.93 | 60.40 | 0.40 | -0.47 | Erecta_GPA1 | 0.51 | 6 | 11.94 | 2.59 |
| CviLerLowFe | Na | 1 | 23.66 | 9.60 | 13.53 | 39.16 | 0.22 | 654.88 | GD.86L | 0.22 | 4 | 11.50 | 2.50 |
| CviLerLowFe | Ni | --- | --- | --- | --- | --- | --- | --- | --- |  | 4 | 10.88 | 2.36 |
| CviLerLowFe | P | 1 | 37.16 | 3.07 | 31.16 | 41.16 | 0.08 | -2246.42 | AD.121C | 0.24 | 8 | 11.94 | 2.59 |
| CviLerLowFe | P | 1 | 127.74 | 2.85 | 125.74 | 127.74 | 0.06 | 2001.16 | GH.127L_Col_ADH | 0.24 | 8 | 11.94 | 2.59 |
| CviLerLowFe | P | 2 | 47.96 | 4.69 | 40.93 | 53.85 | 0.11 | -2681.92 | Erecta_GPA1 | 0.24 | 8 | 11.94 | 2.59 |
| CviLerLowFe | Se | 1 | 15.53 | 2.87 | 15.53 | 17.89 | 0.10 | 0.41 | EC.480C | 0.28 | 6 | 11.66 | 2.53 |
| CviLerLowFe | Se | 2 | 15.59 | 4.37 | 15.59 | 24.22 | 0.18 | -0.67 | GH.580L | 0.28 | 6 | 11.66 | 2.53 |
| CviLerLowFe | Zn | 2 | 69.05 | 2.52 | 67.05 | 69.05 | 0.07 | -21.87 | EC.235L_Col_247C | 0.20 | 8 | 11.22 | 2.44 |
| CviLerLowFe | Zn | 5 | 92.56 | 2.97 | 92.56 | 95.09 | 0.07 | 25.86 | CC.262C | 0.20 | 8 | 11.22 | 2.44 |
| CviLerLowFe | Zn | 5 | 110.59 | 2.79 | 108.59 | 111.92 | 0.06 | -24.00 | DF.119L | 0.20 | 8 | 11.22 | 2.44 |
| BayShaFull | As | 1 | 61.96 | 2.63 | 59.39 | 64.41 | 0.03 | -0.03 | NGA128 | 0.03 | 2 | 10.67 | 2.32 |
| BayShaFull | B | 1 | 2.01 | 6.61 | 0.01 | 13.40 | 0.07 | 3.51 | T1G11 | 0.18 | 8 | 10.98 | 2.38 |
| BayShaFull | B | 1 | 78.95 | 5.83 | 66.72 | 91.59 | 0.06 | -3.43 | MSAT1.13 | 0.18 | 8 | 10.98 | 2.38 |
| BayShaFull | B | 5 | 68.99 | 5.61 | 56.46 | 79.85 | 0.05 | -3.09 | MSAT520037 | 0.18 | 8 | 10.98 | 2.38 |
| BayShaFull | Ca | 1 | 42.66 | 6.25 | 32.11 | 48.66 | 0.04 | 2215.70 | IND1136 | 0.51 | 10 | 11.41 | 2.48 |
| BayShaFull | Ca | 1 | 76.23 | 49.81 | 66.72 | 91.59 | 0.37 | -6144.71 | MSAT1.13 | 0.51 | 10 | 11.41 | 2.48 |
| BayShaFull | Ca | 2 | 47.14 | 11.88 | 44.16 | 66.27 | 0.08 | -2813.84 | MSAT2.7 | 0.51 | 10 | 11.41 | 2.48 |
| BayShaFull | Ca | 4 | 34.59 | 2.65 | 33.12 | 34.59 | 0.01 | 1353.55 | MSAT4.15 | 0.51 | 10 | 11.41 | 2.48 |
| BayShaFull | Cd | 4 | 52.37 | 5.70 | 34.59 | 63.37 | 0.08 | 0.11 | MSAT4.18 | 0.08 | 2 | 10.93 | 2.37 |
| BayShaFull | Co | 1 | 36.11 | 5.89 | 25.35 | 48.66 | 0.05 | -0.02 | IND1136 | 0.27 | 10 | 11.21 | 2.43 |
| BayShaFull | Co | 2 | 8.21 | 3.65 | 4.01 | 10.21 | 0.03 | -0.02 | MSAT200897 | 0.27 | 10 | 11.21 | 2.43 |
| BayShaFull | Co | 3 | 14.29 | 13.87 | 0.01 | 24.29 | 0.16 | 0.04 | MSAT305754 | 0.27 | 10 | 11.21 | 2.43 |
| BayShaFull | Co | 5 | 92.54 | 3.06 | 88.54 | 92.54 | 0.03 | -0.02 | K9I9 | 0.27 | 10 | 11.21 | 2.43 |
| BayShaFull | Cu | 2 | 8.21 | 2.65 | 6.01 | 8.21 | 0.02 | 0.11 | MSAT200897 | 0.22 | 5 | 10.87 | 2.36 |
| BayShaFull | Cu | 3 | 76.14 | 4.33 | 72.14 | 76.14 | 0.04 | -0.14 | MSAT3.70 | 0.22 | 5 | 10.87 | 2.36 |
| BayShaFull | Cu | 5 | 28.79 | 15.07 | 12.29 | 40.35 | 0.16 | -0.27 | NGA139 | 0.22 | 5 | 10.87 | 2.36 |
| BayShaFull | Fe | 1 | 42.66 | 4.72 | 30.26 | 48.66 | 0.04 | 3.37 | IND1136 | 0.31 | 7 | 11.29 | 2.45 |
| BayShaFull | Fe | 1 | 76.95 | 25.65 | 66.72 | 91.59 | 0.22 | -7.63 | MSAT1.13 | 0.31 | 7 | 11.29 | 2.45 |
| BayShaFull | Fe | 2 | 45.14 | 6.93 | 40.16 | 59.74 | 0.05 | -3.82 | MSAT2.7 | 0.31 | 7 | 11.29 | 2.45 |
| BayShaFull | K | 1 | 44.66 | 7.19 | 34.11 | 48.66 | 0.05 | -1633.99 | T27K12 | 0.62 | 10 | 10.66 | 2.31 |
| BayShaFull | K | 1 | 66.41 | 7.64 | 51.62 | 66.41 | 0.05 | -1991.05 | dCAPsAPR2 | 0.62 | 10 | 10.66 | 2.31 |
| BayShaFull | K | 1 | 78.95 | 35.15 | 70.23 | 91.59 | 0.30 | 3733.54 | MSAT1.13 | 0.62 | 10 | 10.66 | 2.31 |
| BayShaFull | K | 2 | 57.74 | 15.65 | 45.14 | 66.27 | 0.12 | 2408.36 | MSAT2.10 | 0.62 | 10 | 10.66 | 2.31 |
| BayShaFull | K | 4 | 10.99 | 4.11 | 6.09 | 14.99 | 0.03 | 1202.12 | MSAT4.43 | 0.62 | 10 | 10.66 | 2.31 |
| BayShaFull | K | 4 | 34.59 | 2.45 | 34.59 | 34.59 | 0.02 | -931.65 | MSAT4.15 | 0.62 | 10 | 10.66 | 2.31 |
| BayShaFull | K | 5 | 82.67 | 7.06 | 70.99 | 92.54 | 0.05 | 1556.06 | JV7576 | 0.62 | 10 | 10.66 | 2.31 |
| BayShaFull | Li | 1 | 89.59 | 2.36 | 89.59 | 89.59 | 0.03 | -1.07 | MSAT1.5 | 0.03 | 5 | 10.82 | 2.35 |
| BayShaFull | Mg | 1 | 44.66 | 9.86 | 38.66 | 48.66 | 0.08 | 449.00 | T27K12 | 0.56 | 10 | 11.02 | 2.39 |
| BayShaFull | Mg | 1 | 78.95 | 42.00 | 66.72 | 91.59 | 0.35 | -908.38 | MSAT1.13 | 0.56 | 10 | 11.02 | 2.39 |
| BayShaFull | Mg | 2 | 59.74 | 9.43 | 28.71 | 66.27 | 0.06 | -366.73 | MSAT2.10 | 0.56 | 10 | 11.02 | 2.39 |
| BayShaFull | Mg | 4 | 0.01 | 3.71 | 0.01 | 0.01 | 0.02 | 231.42 | MSAT4.39 | 0.56 | 10 | 11.02 | 2.39 |
| BayShaFull | Mg | 4 | 8.09 | 3.16 | 4.09 | 10.09 | 0.02 | 240.88 | MSAT4.43 | 0.56 | 10 | 11.02 | 2.39 |
| BayShaFull | Mg | 4 | 38.59 | 3.87 | 31.12 | 44.59 | 0.03 | 262.28 | MSAT4.15 | 0.56 | 10 | 11.02 | 2.39 |
| BayShaFull | Mn | 1 | 36.11 | 7.64 | 32.11 | 44.66 | 0.09 | -4.00 | IND1136 | 0.31 | 10 | 11.00 | 2.39 |
| BayShaFull | Mn | 2 | 51.14 | 2.57 | 49.14 | 55.74 | 0.03 | -1.86 | IND216199 | 0.31 | 10 | 11.00 | 2.39 |
| BayShaFull | Mn | 3 | 16.29 | 9.47 | 6.99 | 24.29 | 0.12 | 3.98 | MSAT305754 | 0.31 | 10 | 11.00 | 2.39 |
| BayShaFull | Mn | 4 | 10.09 | 3.24 | 6.09 | 14.99 | 0.03 | -2.01 | MSAT4.43 | 0.31 | 10 | 11.00 | 2.39 |
| BayShaFull | Mn | 5 | 19.73 | 4.36 | 14.29 | 28.79 | 0.04 | 2.37 | NGA151 | 0.31 | 10 | 11.00 | 2.39 |
| BayShaFull | Mo | 2 | 36.71 | 102.38 | 14.77 | 53.14 | 0.66 | -3.00 | MSAT2.41 | 0.69 | 7 | 11.31 | 2.46 |
| BayShaFull | Mo | 3 | 5.37 | 6.33 | 0.01 | 24.29 | 0.02 | -0.57 | ATHCHIB2 | 0.69 | 7 | 11.31 | 2.46 |
| BayShaFull | Na | 1 | 18.88 | 3.28 | 18.88 | 20.88 | 0.02 | -78.38 | IND6375 | 0.66 | 8 | 11.20 | 2.43 |
| BayShaFull | Na | 1 | 25.35 | 2.50 | 23.35 | 25.35 | 0.02 | -70.58 | MSAT108193 | 0.66 | 8 | 11.20 | 2.43 |
| BayShaFull | Na | 3 | 5.37 | 2.45 | 5.37 | 5.37 | 0.02 | 68.48 | ATHCHIB2 | 0.66 | 8 | 11.20 | 2.43 |
| BayShaFull | Na | 4 | 2.01 | 2.68 | 2.01 | 2.01 | 0.02 | 87.38 | MSAT4.8 | 0.66 | 8 | 11.20 | 2.43 |
| BayShaFull | Na | 4 | 20.12 | 58.06 | 10.99 | 33.12 | 0.56 | -413.17 | NGA8 | 0.66 | 8 | 11.20 | 2.43 |
| BayShaFull | Na | 4 | 57.37 | 2.53 | 57.37 | 57.37 | 0.02 | 76.59 | MSAT4.9 | 0.66 | 8 | 11.20 | 2.43 |
| BayShaFull | Na | 5 | 77.85 | 2.84 | 72.99 | 82.67 | 0.02 | 77.73 | JV6162 | 0.66 | 8 | 11.20 | 2.43 |
| BayShaFull | Ni | 1 | 76.95 | 18.39 | 66.72 | 91.59 | 0.17 | -0.45 | MSAT1.13 | 0.20 | 6 | 10.69 | 2.32 |
| BayShaFull | Ni | 2 | 66.27 | 2.76 | 64.27 | 66.27 | 0.03 | -0.17 | MSAT2.22 | 0.20 | 6 | 10.69 | 2.32 |
| BayShaFull | P | 1 | 5.55 | 11.35 | 0.01 | 20.88 | 0.08 | 348.03 | T1G11 | 0.54 | 10 | 10.84 | 2.35 |
| BayShaFull | P | 1 | 38.66 | 4.16 | 34.11 | 46.66 | 0.03 | 208.69 | IND1136 | 0.54 | 10 | 10.84 | 2.35 |
| BayShaFull | P | 1 | 74.23 | 11.40 | 66.72 | 91.59 | 0.08 | -356.01 | MSAT1.13 | 0.54 | 10 | 10.84 | 2.35 |
| BayShaFull | P | 2 | 32.71 | 7.18 | 20.76 | 44.16 | 0.05 | 269.71 | MSAT2.36 | 0.54 | 10 | 10.84 | 2.35 |
| BayShaFull | P | 3 | 2.01 | 4.06 | 0.01 | 12.29 | 0.03 | 198.79 | MSAT3.99 | 0.54 | 10 | 10.84 | 2.35 |
| BayShaFull | P | 4 | 10.99 | 5.41 | 6.09 | 22.12 | 0.03 | 230.55 | MSAT4.43 | 0.54 | 10 | 10.84 | 2.35 |
| BayShaFull | P | 4 | 63.37 | 8.13 | 48.37 | 69.88 | 0.06 | 294.34 | MSAT4.68 | 0.54 | 10 | 10.84 | 2.35 |
| BayShaFull | P | 5 | 21.73 | 11.56 | 0.01 | 28.79 | 0.09 | -364.03 | NGA151 | 0.54 | 10 | 10.84 | 2.35 |
| BayShaFull | P | 5 | 67.73 | 12.03 | 52.46 | 79.85 | 0.09 | 356.42 | MSAT520037 | 0.54 | 10 | 10.84 | 2.35 |
| BayShaFull | Rb | 1 | 44.66 | 4.45 | 34.11 | 48.66 | 0.04 | -1.34 | T27K12 | 0.53 | 10 | 10.82 | 2.35 |
| BayShaFull | Rb | 1 | 66.41 | 6.77 | 57.39 | 66.41 | 0.05 | -1.96 | dCAPsAPR2 | 0.53 | 10 | 10.82 | 2.35 |
| BayShaFull | Rb | 1 | 78.95 | 21.43 | 70.23 | 91.59 | 0.22 | 3.03 | MSAT1.13 | 0.53 | 10 | 10.82 | 2.35 |
| BayShaFull | Rb | 2 | 57.74 | 12.96 | 44.16 | 66.27 | 0.14 | 2.39 | MSAT2.10 | 0.53 | 10 | 10.82 | 2.35 |
| BayShaFull | Rb | 4 | 8.09 | 2.42 | 8.09 | 10.09 | 0.02 | 0.95 | MSAT4.43 | 0.53 | 10 | 10.82 | 2.35 |
| BayShaFull | Rb | 5 | 38.35 | 2.43 | 36.35 | 40.35 | 0.02 | 0.99 | MSAT512110 | 0.53 | 10 | 10.82 | 2.35 |
| BayShaFull | Rb | 5 | 82.67 | 4.19 | 72.99 | 90.54 | 0.04 | 1.24 | JV7576 | 0.53 | 10 | 10.82 | 2.35 |
| BayShaFull | S | 1 | 66.41 | 70.34 | 55.39 | 76.23 | 0.29 | 1602.29 | dCAPsAPR2 | 0.69 | 10 | 11.01 | 2.39 |
| BayShaFull | S | 3 | 10.29 | 50.48 | 0.01 | 41.73 | 0.23 | -1421.90 | MSAT305754 | 0.69 | 10 | 11.01 | 2.39 |
| BayShaFull | S | 3 | 76.14 | 8.26 | 62.94 | 76.14 | 0.02 | 469.97 | MSAT3.70 | 0.69 | 10 | 11.01 | 2.39 |
| BayShaFull | S | 4 | 12.99 | 19.89 | 0.01 | 44.59 | 0.07 | -805.37 | MSAT4.43 | 0.69 | 10 | 11.01 | 2.39 |
| BayShaFull | S | 4 | 50.37 | 2.78 | 48.34 | 52.37 | 0.01 | -330.31 | MSAT4.18 | 0.69 | 10 | 11.01 | 2.39 |
| BayShaFull | S | 5 | 26.79 | 12.55 | 12.29 | 36.35 | 0.04 | 688.55 | MSAT5.14 | 0.69 | 10 | 11.01 | 2.39 |
| BayShaFull | S | 5 | 42.10 | 2.49 | 42.10 | 42.10 | 0.01 | -299.48 | MSAT512110 | 0.69 | 10 | 11.01 | 2.39 |
| BayShaFull | S | 5 | 79.85 | 6.31 | 68.99 | 88.54 | 0.02 | -433.05 | JV7576 | 0.69 | 10 | 11.01 | 2.39 |
| BayShaFull | Se | 1 | 68.72 | 8.78 | 57.39 | 76.23 | 0.09 | 2.12 | F5I14 | 0.25 | 5 | 10.76 | 2.34 |
| BayShaFull | Se | 3 | 2.01 | 2.65 | 0.01 | 3.37 | 0.03 | -1.25 | MSAT3.99 | 0.25 | 5 | 10.76 | 2.34 |
| BayShaFull | Se | 3 | 16.29 | 5.12 | 8.29 | 24.29 | 0.07 | -1.85 | MSAT305754 | 0.25 | 5 | 10.76 | 2.34 |
| BayShaFull | Se | 4 | 25.12 | 4.09 | 10.99 | 31.12 | 0.04 | -1.38 | MSAT4.35 | 0.25 | 5 | 10.76 | 2.34 |
| BayShaFull | Se | 4 | 44.59 | 2.51 | 40.59 | 44.59 | 0.03 | -1.18 | CIW7 | 0.25 | 5 | 10.76 | 2.34 |
| BayShaFull | Zn | 1 | 66.41 | 2.76 | 66.41 | 66.41 | 0.03 | -2.50 | dCAPsAPR2 | 0.23 | 10 | 11.01 | 2.39 |
| BayShaFull | Zn | 3 | 74.14 | 6.60 | 62.94 | 76.14 | 0.07 | -3.11 | MSAT3.70 | 0.23 | 10 | 11.01 | 2.39 |
| BayShaFull | Zn | 4 | 33.12 | 4.13 | 22.12 | 40.59 | 0.04 | 2.42 | MSAT4.15 | 0.23 | 10 | 11.01 | 2.39 |
| BayShaFull | Zn | 4 | 65.88 | 3.30 | 61.37 | 69.88 | 0.03 | -2.25 | MSAT4.68 | 0.23 | 10 | 11.01 | 2.39 |
| BayShaFull | Zn | 5 | 84.67 | 6.13 | 75.85 | 92.54 | 0.06 | -2.96 | MSAT5.19 | 0.23 | 10 | 11.01 | 2.39 |
| BayShaSmall | As | --- | --- | --- | --- | --- | --- | --- | --- | --- | 2 | 11.81 | 2.56 |
| BayShaSmall | B | 1 | 7.55 | 3.08 | 5.55 | 7.55 | 0.07 | 16.57 | F21M12 | 0.20 | 9 | 10.31 | 2.24 |
| BayShaSmall | B | 1 | 76.95 | 3.42 | 72.23 | 80.95 | 0.07 | -13.70 | MSAT1.13 | 0.20 | 9 | 10.31 | 2.24 |
| BayShaSmall | B | 5 | 14.29 | 2.42 | 14.29 | 17.73 | 0.06 | 12.49 | NGA249 | 0.20 | 9 | 10.31 | 2.24 |
| BayShaSmall | Ca | 1 | 40.66 | 4.99 | 34.11 | 48.66 | 0.09 | 1572.37 | IND1136 | 0.61 | 6 | 11.29 | 2.45 |
| BayShaSmall | Ca | 1 | 76.95 | 22.45 | 66.72 | 91.59 | 0.42 | -3455.48 | MSAT1.13 | 0.61 | 6 | 11.29 | 2.45 |
| BayShaSmall | Ca | 2 | 47.14 | 3.58 | 40.16 | 51.14 | 0.06 | -1303.89 | MSAT2.7 | 0.61 | 6 | 11.29 | 2.45 |
| BayShaSmall | Ca | 4 | 22.12 | 2.58 | 20.12 | 24.12 | 0.04 | 1069.14 | MSAT4.35 | 0.61 | 6 | 11.29 | 2.45 |
| BayShaSmall | Cd111 | 4 | 54.37 | 3.89 | 50.37 | 56.37 | 0.11 | 0.11 | MSAT4.9 | 0.11 | 7 | 11.40 | 2.47 |
| BayShaSmall | Co | 3 | 22.29 | 2.85 | 16.29 | 26.14 | 0.10 | 0.06 | MSAT3.19 | 0.10 | 4 | 10.89 | 2.36 |
| BayShaSmall | Cu | 1 | 9.40 | 3.51 | 3.55 | 13.40 | 0.07 | -0.40 | F21M12 | 0.38 | 8 | 11.42 | 2.48 |
| BayShaSmall | Cu | 3 | 22.29 | 2.54 | 22.29 | 24.29 | 0.07 | -0.40 | MSAT3.19 | 0.38 | 8 | 11.42 | 2.48 |
| BayShaSmall | Cu | 3 | 59.21 | 4.12 | 44.77 | 66.94 | 0.09 | 0.45 | MSAT318406 | 0.38 | 8 | 11.42 | 2.48 |
| BayShaSmall | Cu | 4 | 69.88 | 3.23 | 67.88 | 69.88 | 0.07 | -0.41 | MSAT4.37 | 0.38 | 8 | 11.42 | 2.48 |
| BayShaSmall | Cu | 5 | 19.73 | 4.09 | 17.73 | 28.79 | 0.09 | -0.49 | NGA151 | 0.38 | 8 | 11.42 | 2.48 |
| BayShaSmall | Fe56 | 4 | 12.99 | 2.78 | 6.09 | 16.12 | 0.07 | 1.74 | MSAT4.43 | 0.07 | 6 | 11.63 | 2.52 |
| BayShaSmall | K | 1 | 15.17 | 6.61 | 5.55 | 23.35 | 0.07 | -2253.04 | IND4992 | 0.84 | 10 | 10.93 | 2.37 |
| BayShaSmall | K | 1 | 44.66 | 8.72 | 38.66 | 48.66 | 0.12 | -3019.85 | T27K12 | 0.84 | 10 | 10.93 | 2.37 |
| BayShaSmall | K | 1 | 76.23 | 25.67 | 66.72 | 91.59 | 0.37 | 5350.22 | MSAT1.13 | 0.84 | 10 | 10.93 | 2.37 |
| BayShaSmall | K | 2 | 57.74 | 8.85 | 45.14 | 66.27 | 0.11 | 2785.29 | MSAT2.10 | 0.84 | 10 | 10.93 | 2.37 |
| BayShaSmall | K | 3 | 5.37 | 2.69 | 2.01 | 6.99 | 0.03 | 1393.83 | ATHCHIB2 | 0.84 | 10 | 10.93 | 2.37 |
| BayShaSmall | K | 4 | 18.12 | 3.19 | 10.99 | 22.12 | 0.04 | 1680.89 | NGA8 | 0.84 | 10 | 10.93 | 2.37 |
| BayShaSmall | K | 4 | 50.37 | 5.69 | 36.59 | 56.37 | 0.07 | -2439.90 | MSAT4.18 | 0.84 | 10 | 10.93 | 2.37 |
| BayShaSmall | K | 5 | 77.85 | 3.65 | 72.99 | 88.54 | 0.04 | 1704.14 | JV6162 | 0.84 | 10 | 10.93 | 2.37 |
| BayShaSmall | Li | 2 | 8.21 | 3.36 | 4.01 | 10.21 | 0.08 | 0.26 | MSAT200897 | 0.15 | 5 | 11.05 | 2.40 |
| BayShaSmall | Li | 5 | 92.54 | 2.81 | 92.54 | 92.54 | 0.07 | 0.23 | K9I9 | 0.15 | 5 | 11.05 | 2.40 |
| BayShaSmall | Mg | 1 | 38.66 | 6.17 | 34.11 | 48.66 | 0.09 | 401.43 | IND1136 | 0.46 | 10 | 11.06 | 2.40 |
| BayShaSmall | Mg | 1 | 76.23 | 20.45 | 66.72 | 91.59 | 0.37 | -801.68 | MSAT1.13 | 0.46 | 10 | 11.06 | 2.40 |
| BayShaSmall | Mn | 1 | 91.59 | 4.27 | 82.95 | 91.59 | 0.10 | 2.75 | MSAT1.5 | 0.20 | 5 | 11.53 | 2.50 |
| BayShaSmall | Mn | 3 | 48.77 | 3.97 | 42.77 | 59.21 | 0.10 | 2.77 | MSAT3.21 | 0.20 | 5 | 11.53 | 2.50 |
| BayShaSmall | Mo | 2 | 37.13 | 64.70 | 14.77 | 53.14 | 0.80 | -5.29 | MSAT2.41 | 0.81 | 5 | 11.75 | 2.55 |
| BayShaSmall | Mo | 3 | 3.37 | 3.94 | 2.01 | 6.99 | 0.02 | -0.79 | MSAT3.99 | 0.81 | 5 | 11.75 | 2.55 |
| BayShaSmall | Na | 4 | 20.12 | 34.56 | 10.09 | 33.12 | 0.61 | -214.31 | NGA8 | 0.66 | 8 | 11.31 | 2.46 |
| BayShaSmall | Na | 5 | 19.73 | 3.41 | 14.29 | 23.73 | 0.05 | 64.10 | NGA151 | 0.66 | 8 | 11.31 | 2.46 |
| BayShaSmall | Ni | --- | --- | --- | --- | --- | --- | --- | --- | --- | 3 | 11.68 | 2.54 |
| BayShaSmall | P | 1 | 2.01 | 4.08 | 0.01 | 7.55 | 0.05 | 302.73 | T1G11 | 0.72 | 10 | 10.98 | 2.38 |
| BayShaSmall | P | 1 | 66.72 | 3.65 | 63.96 | 68.72 | 0.04 | -277.70 | dCAPsAPR2 | 0.72 | 10 | 10.98 | 2.38 |
| BayShaSmall | P | 2 | 10.21 | 6.43 | 0.01 | 13.11 | 0.10 | -462.37 | MSAT200897 | 0.72 | 10 | 10.98 | 2.38 |
| BayShaSmall | P | 2 | 32.71 | 10.95 | 22.76 | 39.13 | 0.16 | 555.89 | MSAT2.36 | 0.72 | 10 | 10.98 | 2.38 |
| BayShaSmall | P | 3 | 8.29 | 4.86 | 0.01 | 18.29 | 0.06 | 314.32 | MSAT305754 | 0.72 | 10 | 10.98 | 2.38 |
| BayShaSmall | P | 5 | 21.73 | 8.19 | 0.01 | 28.79 | 0.13 | -518.11 | NGA151 | 0.72 | 10 | 10.98 | 2.38 |
| BayShaSmall | P | 5 | 63.73 | 12.55 | 52.46 | 75.22 | 0.17 | 551.83 | MSAT518662 | 0.72 | 10 | 10.98 | 2.38 |
| BayShaSmall | Se | 1 | 53.62 | 2.58 | 53.62 | 53.62 | 0.04 | -0.70 | MSAT1.42 | 0.56 | 8 | 11.75 | 2.55 |
| BayShaSmall | Se | 1 | 66.72 | 18.08 | 57.39 | 74.23 | 0.27 | 1.99 | dCAPsAPR2 | 0.56 | 8 | 11.75 | 2.55 |
| BayShaSmall | Se | 3 | 14.29 | 13.16 | 0.01 | 35.73 | 0.24 | -1.44 | MSAT305754 | 0.56 | 8 | 11.75 | 2.55 |
| BayShaSmall | Zn | 1 | 3.55 | 2.65 | 3.55 | 5.55 | 0.04 | -1.94 | T1G11 | 0.39 | 9 | 11.38 | 2.47 |
| BayShaSmall | Zn | 1 | 11.40 | 4.87 | 9.40 | 13.40 | 0.09 | -2.60 | F21M12 | 0.39 | 9 | 11.38 | 2.47 |
| BayShaSmall | Zn | 1 | 91.59 | 4.64 | 85.59 | 91.59 | 0.08 | 2.54 | MSAT1.5 | 0.39 | 9 | 11.38 | 2.47 |
| BayShaSmall | Zn | 3 | 44.77 | 3.94 | 37.73 | 51.77 | 0.07 | 2.40 | MSAT3.32 | 0.39 | 9 | 11.38 | 2.47 |
| BayShaSmall | Zn | 4 | 25.12 | 3.16 | 22.12 | 29.12 | 0.05 | 1.96 | MSAT4.35 | 0.39 | 9 | 11.38 | 2.47 |
| BayShaSmall | Zn | 5 | 21.73 | 3.38 | 14.29 | 26.79 | 0.06 | -2.30 | NGA151 | 0.39 | 9 | 11.38 | 2.47 |
| ColLer | B | 1 | 12.03 | 2.88 | 11.14 | 16.03 | 0.13 | 6.95 | C1_21 | 0.13 | 1 | 11.11 | 2.41 |
| ColLer | Ca | --- | --- | --- | --- | --- | --- | --- | --- |  | 3 | 11.60 | 2.52 |
| ColLer | Cd | 2 | 71.05 | 3.69 | 67.05 | 71.05 | 0.13 | -0.56 | C2_106 | 0.36 | 6 | 11.62 | 2.52 |
| ColLer | Cd | 4 | 17.15 | 3.78 | 12.40 | 19.15 | 0.12 | -0.71 | C4_19 | 0.36 | 6 | 11.62 | 2.52 |
| ColLer | Cd | 5 | 114.12 | 3.61 | 114.12 | 114.12 | 0.11 | -0.74 | C5_164 | 0.36 | 6 | 11.62 | 2.52 |
| ColLer | Co | --- | --- | --- | --- | --- | --- | --- | --- |  | 3 | 11.39 | 2.47 |
| ColLer | Cu | --- | --- | --- | --- | --- | --- | --- | --- |  | 2 | 11.32 | 2.46 |
| ColLer | Fe | 1 | 7.54 | 4.07 | 3.54 | 11.14 | 0.19 | -4.08 | C1_14 | 0.19 | 4 | 11.09 | 2.41 |
| ColLer | K | 3 | 56.11 | 3.03 | 54.64 | 58.11 | 0.12 | -2326.91 | C3_76 | 0.22 | 3 | 11.48 | 2.49 |
| ColLer | K | 4 | 19.42 | 2.63 | 19.15 | 19.42 | 0.10 | 2192.24 | C4_22 | 0.22 | 3 | 11.48 | 2.49 |
| ColLer | Li | --- | --- | --- | --- | --- | --- | --- | --- |  | 1 | 11.55 | 2.51 |
| ColLer | Mg | --- | --- | --- | --- | --- | --- | --- | --- |  | 1 | 11.41 | 2.48 |
| ColLer | Mn | --- | --- | --- | --- | --- | --- | --- | --- |  | 3 | 11.48 | 2.49 |
| ColLer | Mo | 2 | 45.27 | 2.63 | 39.27 | 47.80 | 0.12 | 0.49 | C2_67 | 0.12 | 4 | 10.00 | 2.17 |
| ColLer | Mo | 4 | 19.15 | 2.44 | 19.15 | 19.15 | 0.11 | 0.66 | C4_22 | 0.11 | 4 | 10.00 | 2.17 |
| ColLer | Na | --- | --- | --- | --- | --- | --- | --- | --- |  | 3 | 11.01 | 2.39 |
| ColLer | Ni | 4 | 30.01 | 1.87 | 26.48 | 32.01 | 0.08 | 0.13 | C4_40 | 0.08 | 3 | 7.78 | 1.69 |
| ColLer | P | 1 | 0.01 | 3.72 | 0.00 | 2.01 | 0.15 | -639.23 | C1_1 | 0.15 | 3 | 11.28 | 2.45 |
| ColLer | Se | --- | --- | --- | --- | --- | --- | --- | --- |  | 1 | 10.90 | 2.37 |
| ColLer | Zn | --- | --- | --- | --- | --- | --- | --- | --- |  | 2 | 11.13 | 2.42 |
