# Supplementary material for: Natural Genetic Variation in Selected Populations of Arabidopsis thaliana Is Associated with Ionomic Differences
Source: PLoS One. 2010 Jun 14;5(6):e11081. doi: 10.1371/journal.pone.0011081 (PMC2885407; doi:10.1371/journal.pone.0011081)
Supplement: Table S3 — Two-way epistatic interactions for each RIL population across all 5 chromosomes. Lod.full is the log-odds ratio of the full model with two loci and their interaction compared to the null model with no QTL. Lod.fv1 is the log-odds ratio of the full model compared to the best single QTL model with one locus on either chromosome A or B (not necessarily at the same location as the full model loci). Lod.int is the log-odds ratio of the interaction term which is found by comparing the full model with an interaction term, to the two QTL model with no interaction term. Lod.add is the log-odds ratio of the additive effects, found by comparing the two QTL model (no interaction term) to the null model with no QTL. Lod.av1 is the log-odds ratio comparing the two QTL model with no interaction term, to the best single QTL model with one locus on either chromosome A or B (not necessarily at the same location as in the two QTL model). (0.04 MB DOC) [file pone.0011081.s003.doc]

|  |  |  |  |  |  |  |  |  |  |  |  |  |
| --- | --- | --- | --- | --- | --- | --- | --- | --- | --- | --- | --- | --- |
|  | **Supplemental table 3: Epistatic interactions determind within the populations** | | | | | | | | | |  |  |
|  |  |  | Loci 1 | | Loci 2 | |  |  |  |  |  |  |
|  | Population | Element | Chr | Pos (cM) | Chr | Pos (cM) | lod.full | lod.fv1 | lod.int | lod.add | lod.av1 |  |
|  | CviLerhigh | K | 1 | 18 | 5 | 28 | 6.46 | 0.53 | 4.37 | 2.09 | -3.84 |  |
|  | CviLerhigh | Mn | 1 | 28 | 3 | 22 | 5.87 | -0.75 | 4.87 | 1.00 | -5.62 |  |
|  | CviLerhigh | Mn | 1 | 18 | 5 | 28 | 8.37 | 1.76 | 4.15 | 4.23 | -2.39 |  |
|  | CviLerhigh | Cu | 3 | 68 | 5 | 96 | 5.06 | 3.44 | 4.42 | 0.64 | -0.98 |  |
|  | CviLerlow | Se | 1 | 1 | 5 | 34 | 8.98 | 6.90 | 6.52 | 2.47 | 0.38 |  |
|  |  |  |  |  |  |  |  |  |  |  |  |  |
|  |  |  |  |  |  |  |  |  |  |  |  |  |
|  |  |  |  |  |  |  |  |  |  |  |  |  |
|  |  |  |  |  |  |  |  |  |  |  |  |  |
|  |  |  |  |  |  |  |  |  |  |  |  |  |
|  |  |  |  |  |  |  |  |  |  |  |  |  |
|  |  |  |  |  |  |  |  |  |  |  |  |  |
