# Supplementary material for: Natural Genetic Variation in Selected Populations of Arabidopsis thaliana Is Associated with Ionomic Differences
Source: PLoS One. 2010 Jun 14;5(6):e11081. doi: 10.1371/journal.pone.0011081 (PMC2885407; doi:10.1371/journal.pone.0011081)
Supplement: Table S4 — Results of in silico experimental design simulations. A. Number of QTLs detected in the subset of CviLer lines with 3 samples analyzed. First two rows indicate the average of 10 randomly generated subsets with n = 1 or 2, third row indicates the number of QTLs identified with the full n = 3 dataset. B. QTLs identified from the large BaySha experiment when either the full 411 lines or the subset of 165 lines corresponding to the smaller BaySha set was used. (0.05 MB DOC) [file pone.0011081.s004.doc]

| 4A | n | Li | B | Na | Mg | P | K | Ca | Mn | Fe | Co | Ni | Cu | Zn | As | Se | Mo | Cd | total |  |  |
| --- | --- | --- | --- | --- | --- | --- | --- | --- | --- | --- | --- | --- | --- | --- | --- | --- | --- | --- | --- | --- | --- |
|  | 1 | 1.7 | 3.1 | 1.8 | 1.6 | 4.9 | 1.4 | 1 | 2 | 1.7 | 1.1 | 1 | 1.3 | 4.5 | 1.1 | 1 | 1.5 | 2.9 | 31.9 |  |  |
|  | 2 | 2.9 | 4.9 | 2.9 | 3.3 | 5.7 | 1.4 | 1.4 | 2.9 | 3 | 1.1 | 1 | 2.6 | 5 | 1.6 | 1.2 | 1.2 | 4.5 | 43.7 |  |  |
|  | 3 | 2 | 5 | 3 | 3 | 6 | 2 | 3 | 2 | 6 | 2 | 1 | 1 | 5 | 1 | 1 | 2 | 4 | 47 |  |  |
|  |  |  |  |  |  |  |  |  |  |  |  |  |  |  |  |  |  |  |  |  |  |
|  |  |  |  |  |  |  |  |  |  |  |  |  |  |  |  |  |  |  |  |  |  |
| 4B |  | Li | B | Na | Mg | P | S | K | Ca | Mn | Fe | Co | Ni | Cu | Zn | As | Se | Rb | Mo | Cd | total |
|  | 411 lines | 1 | 3 | 7 | 6 | 9 | 8 | 7 | 4 | 5 | 3 | 4 | 2 | 3 | 5 | 1 | 5 | 7 | 2 | 1 | 82 |
|  | 165 lines | 1 | 1 | 1 | 5 | 8 | 9 | 7 | 5 | 3 | 2 | 3 | 3 | 1 | 6 | 0 | 2 | 4 | 2 | 2 | 64 |
