# Supplementary figures and images for: Natural Genetic Variation in Selected Populations of Arabidopsis thaliana Is Associated with Ionomic Differences
Source: PLoS One. 2010 Jun 14;5(6):e11081. doi: 10.1371/journal.pone.0011081 (PMC2885407; doi:10.1371/journal.pone.0011081)

### Li7

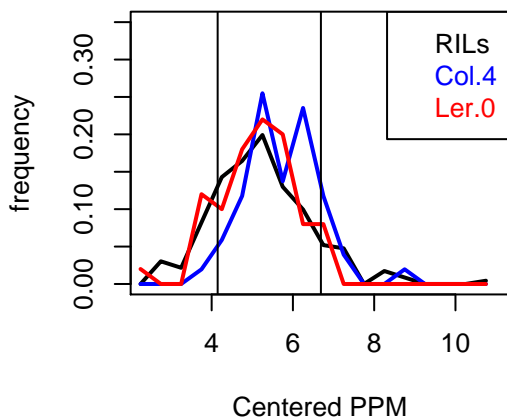

### B11

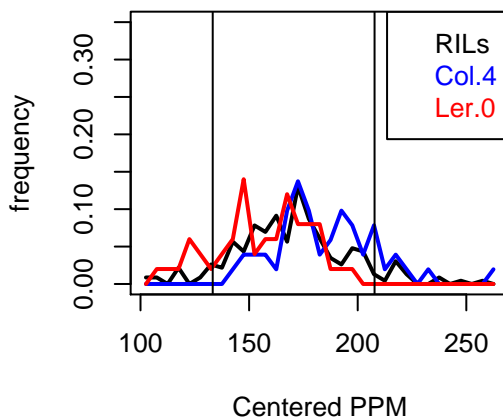

### Na23

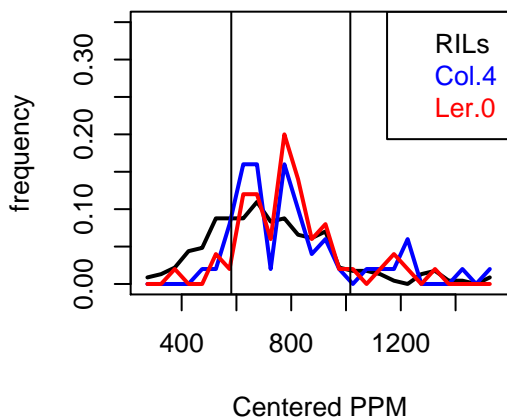

### Mg25

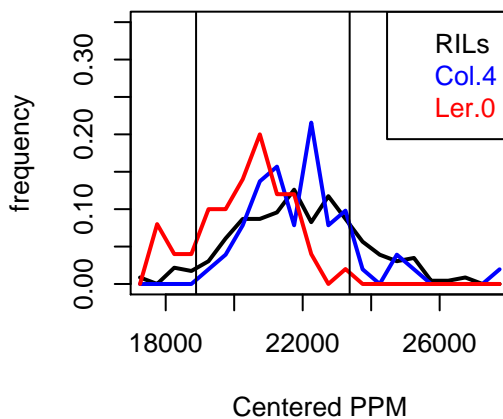

**P31**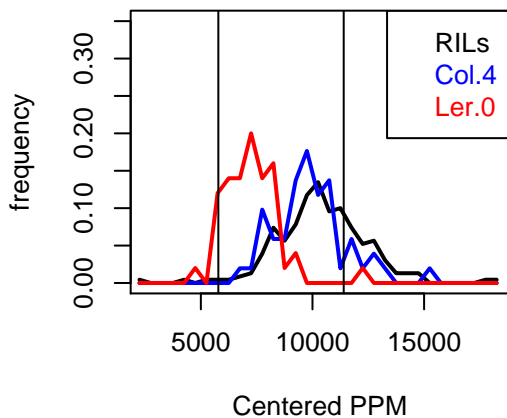**K39**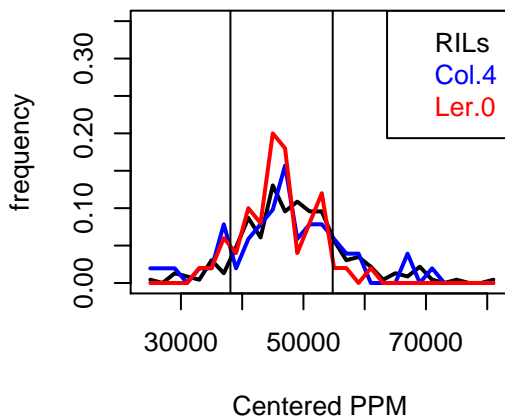**Ca43**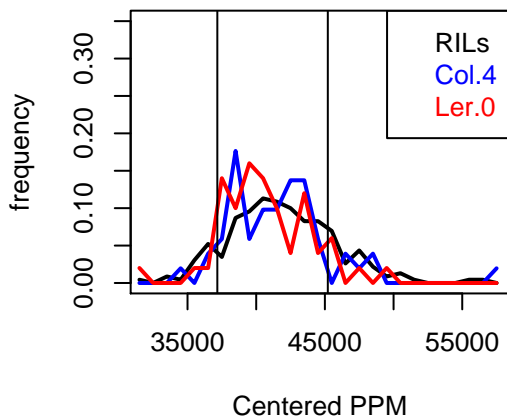**Mn55**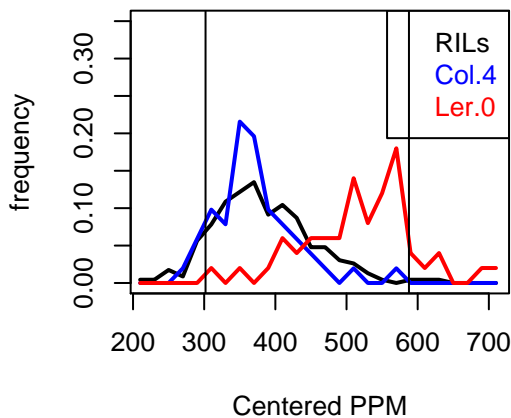

**Fe56**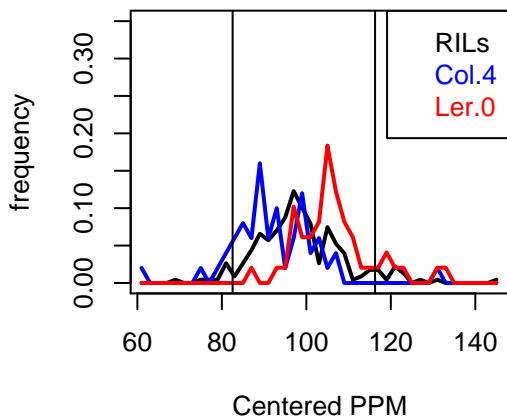**Co59**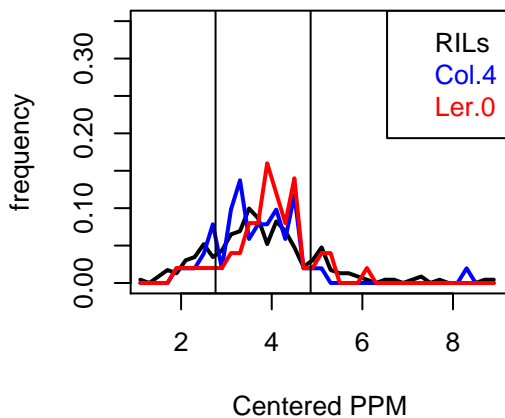**Ni60**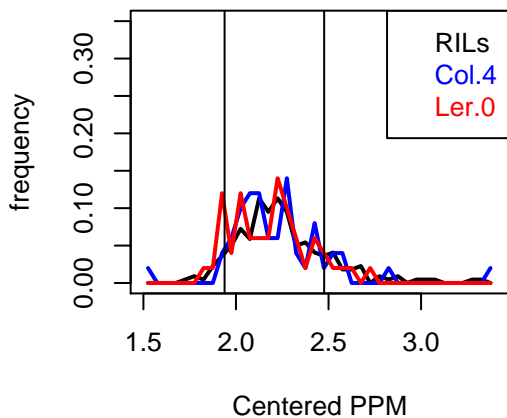**Cu65**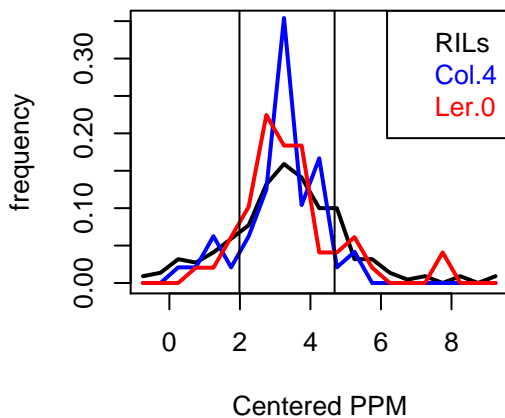

**Zn66**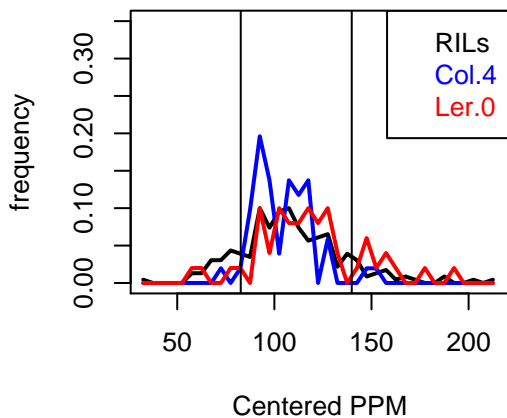**As75**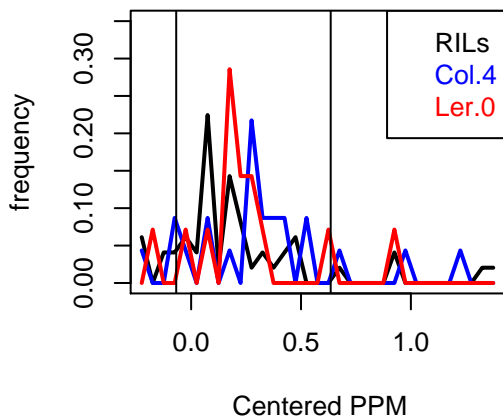**Se77**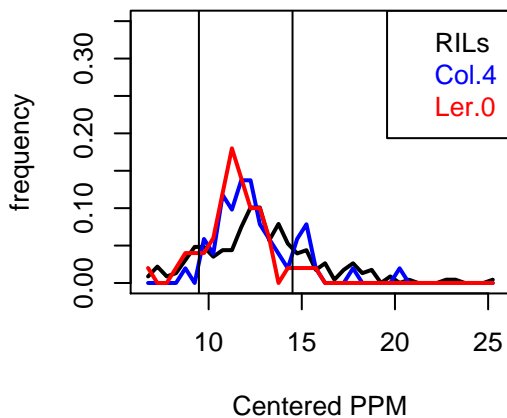**Mo95**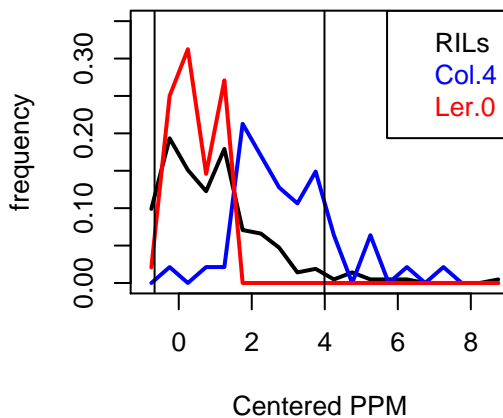

# Cd111

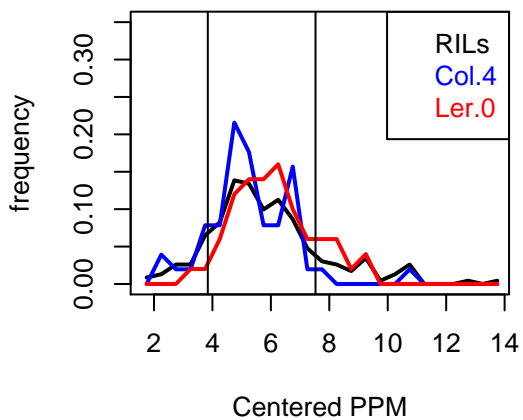

Supplement: File S1 — Frequency plots of parental lines and RILs for each element across the 5 RIL populations. X-axis represents the centered PPM (See Methods) of indicated element. Y-axis indicates frequency of occurrence. Black vertical lines indicate the 95% confidence interval of the parents distribution (i.e. lower parent−1.96 SD (pooled) to higher parent+1.96 SD(pooled)) 1.1. Comparison of ColLer. (0.06 MB PDF) [file pone.0011081.s005.pdf]

**Li7**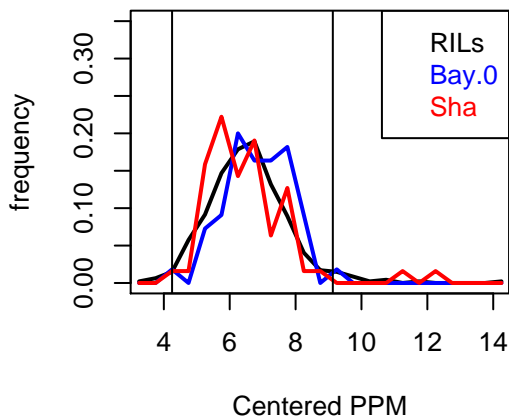**B11**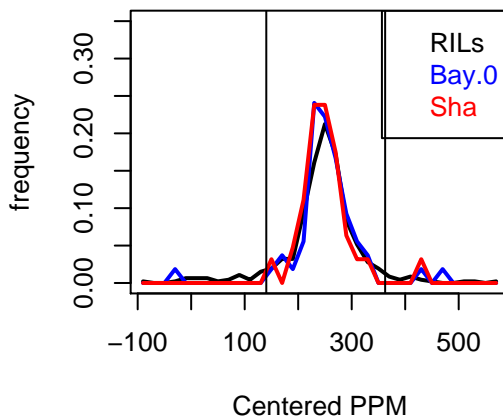**Na23**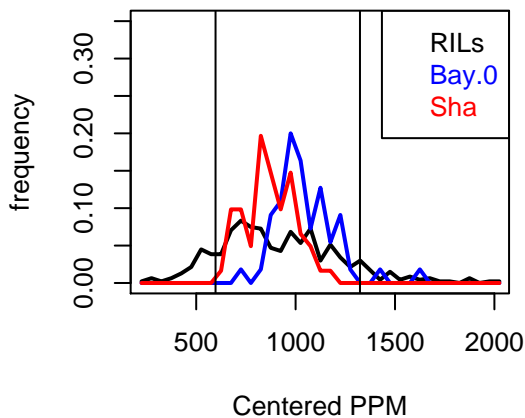**Mg25**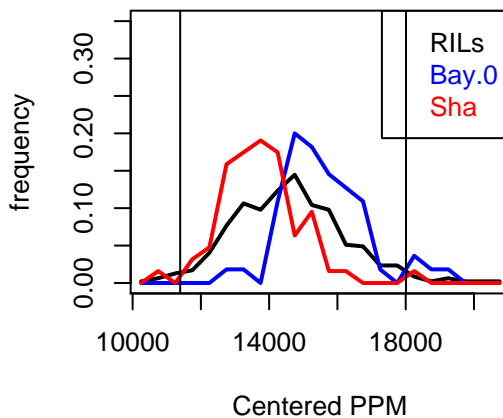

**P31**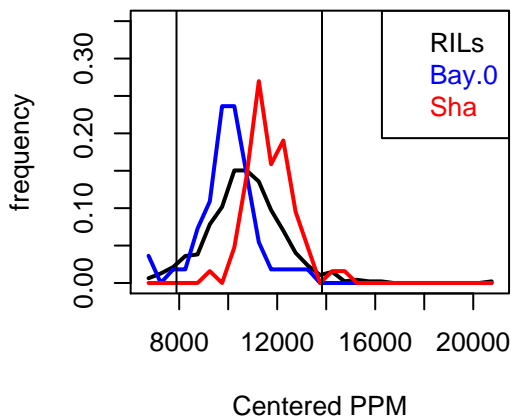**K39**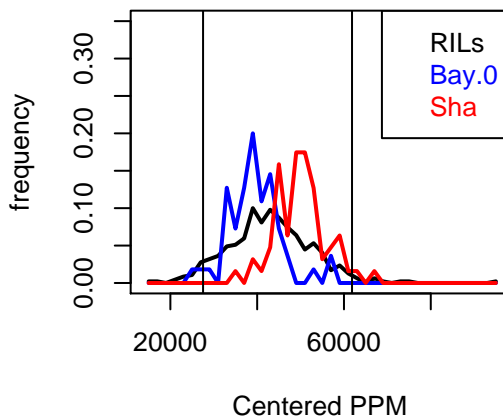**Ca43**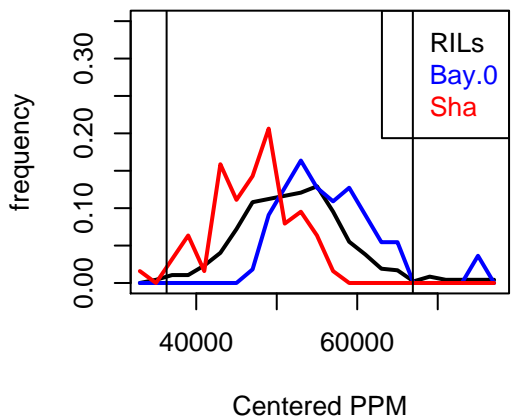**Mn55**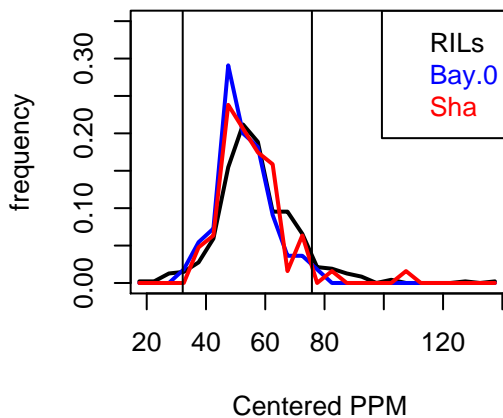

**Fe56**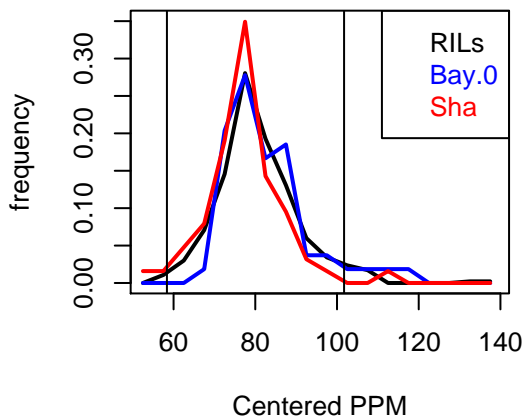**Co59**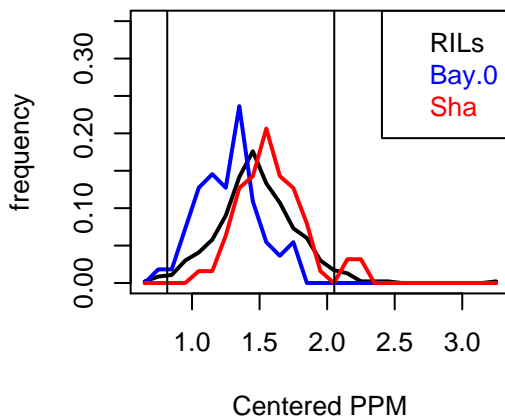**Ni60**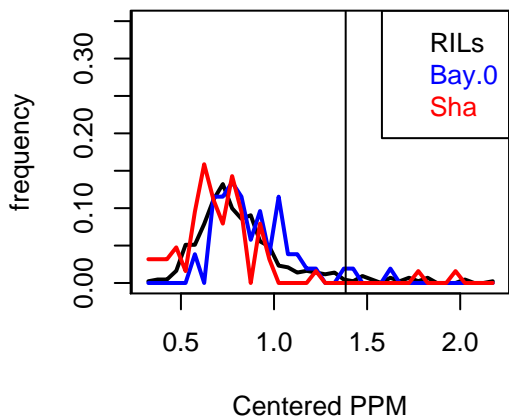**Cu65**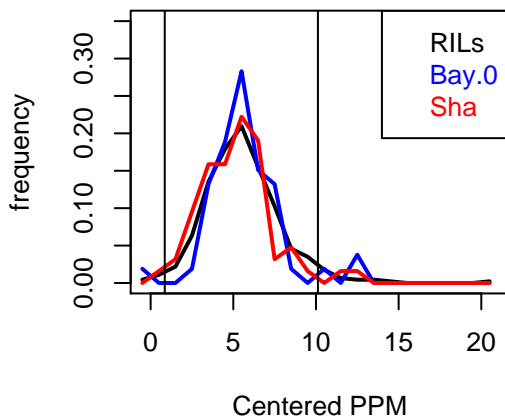

**Zn66**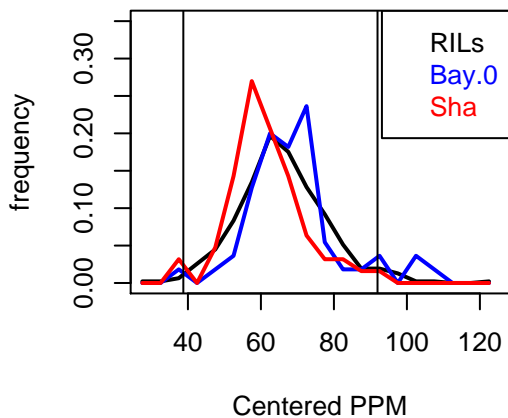**As75**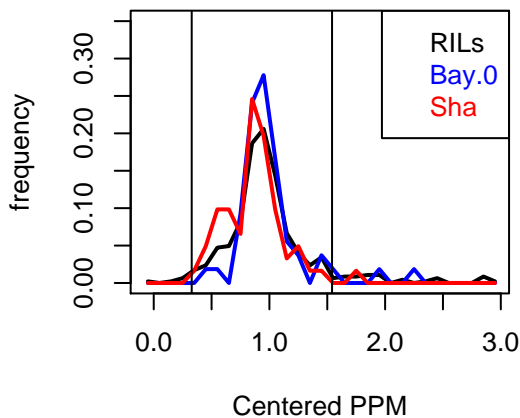**Se77**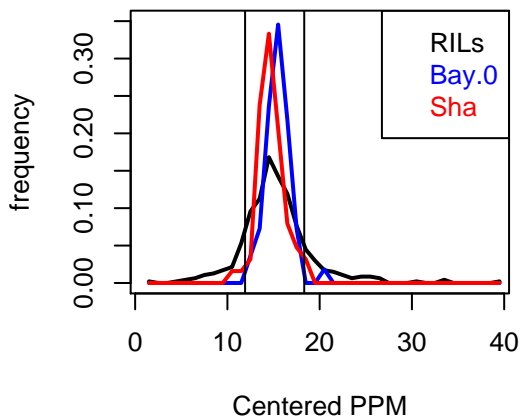**Mo95**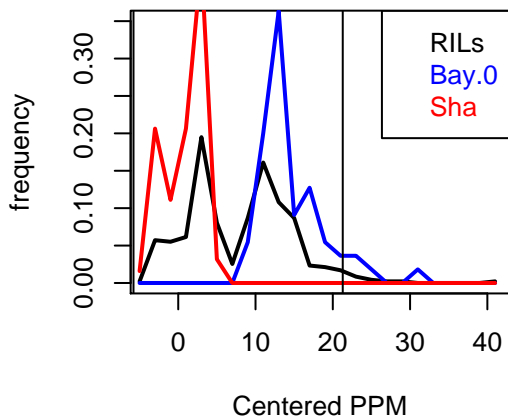

# Cd111

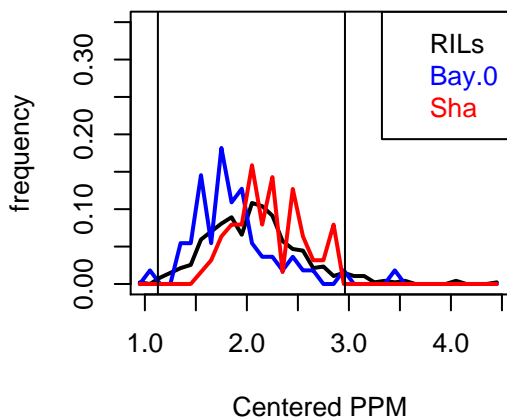

Supplement: File S2 — Frequency plots of parental lines and RILs for each element across the 5 RIL populations. X-axis represents the centered PPM (See Methods) of indicated element. Y-axis indicates frequency of occurrence. Black vertical lines indicate the 95% confidence interval of the parents distribution (i.e. lower parent−1.96 SD (pooled) to higher parent+1.96 SD(pooled)) 2. Comparison of BaySha, grown in Sunshine Soil. (0.06 MB PDF) [file pone.0011081.s006.pdf]

**Li7**

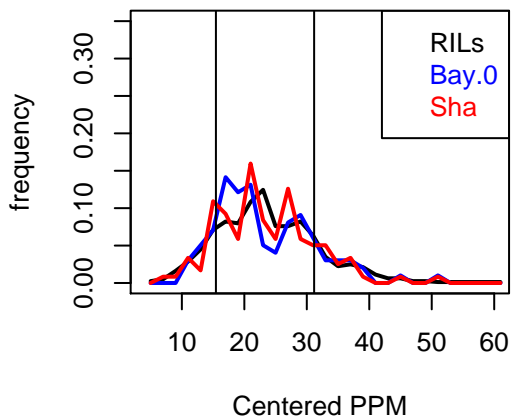

**B11**

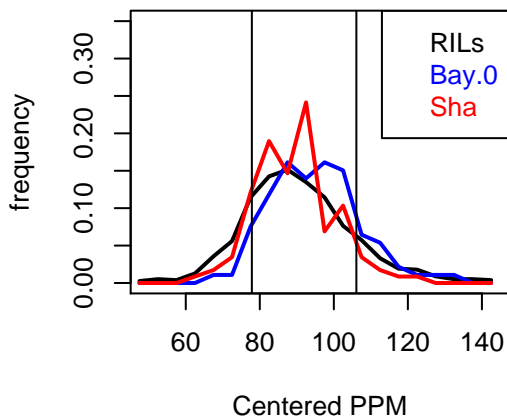

**Na23**

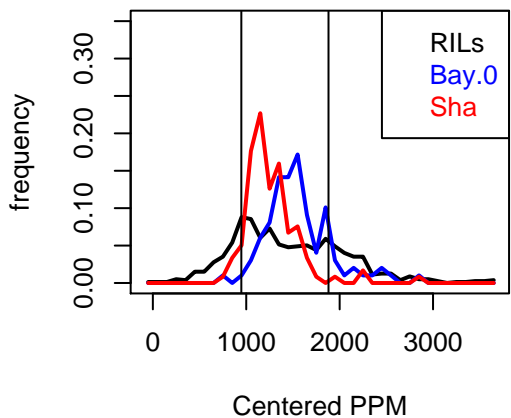

**Mg25**

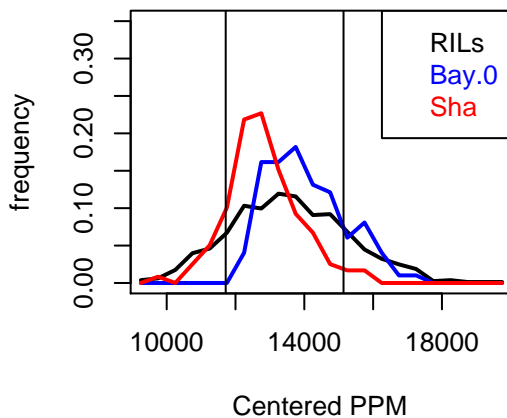

**P31**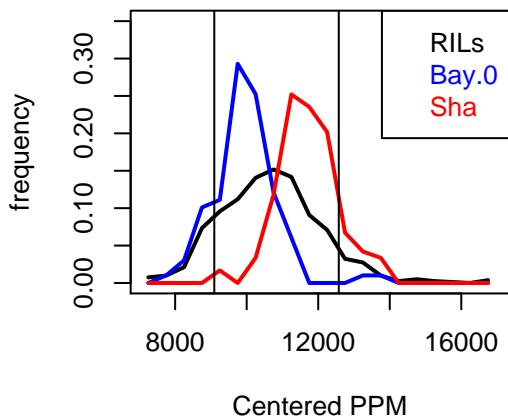**S34**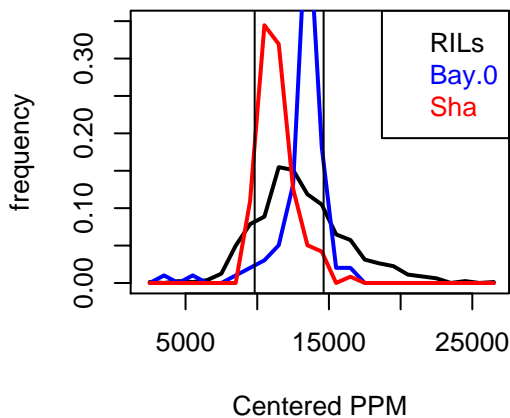**K39**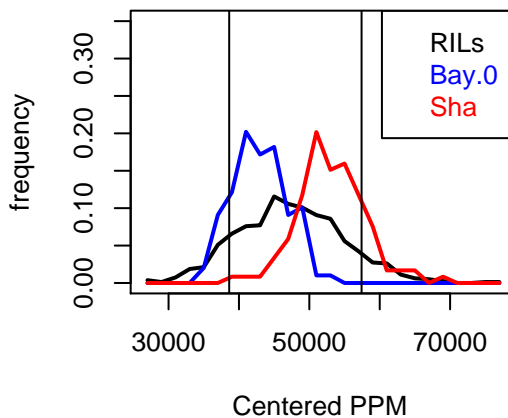**Ca43**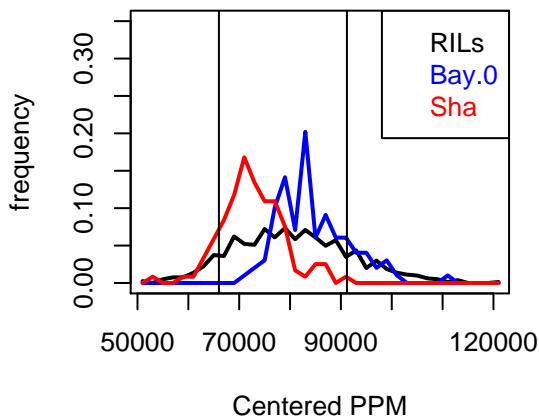

**Mn55**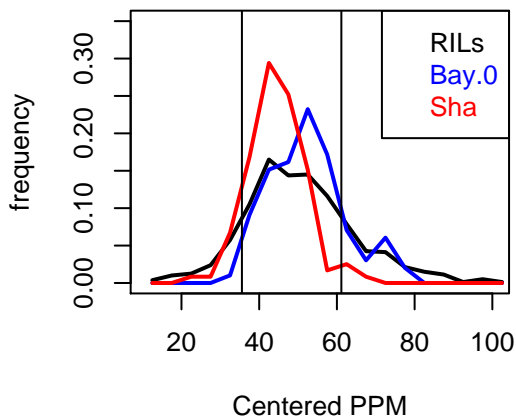**Fe57**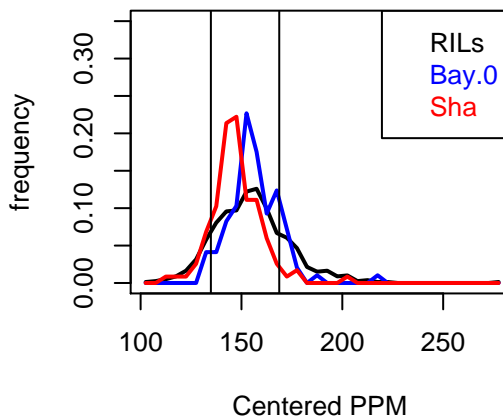**Co59**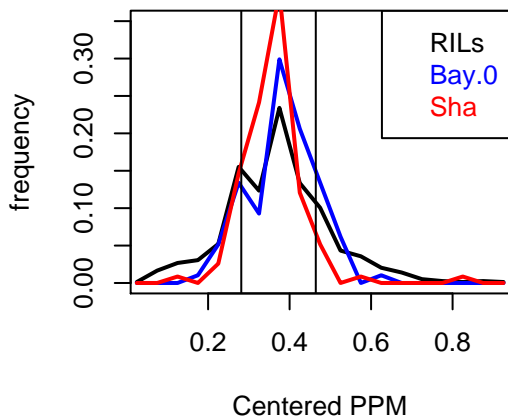**Ni60**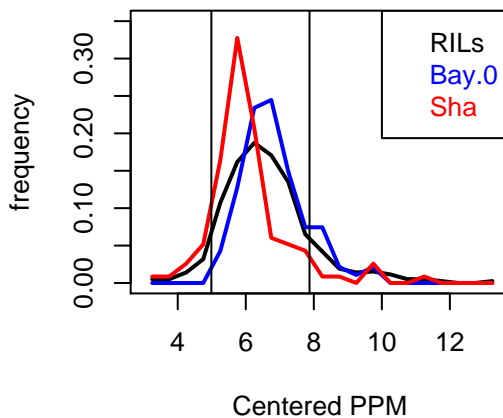

**Cu65**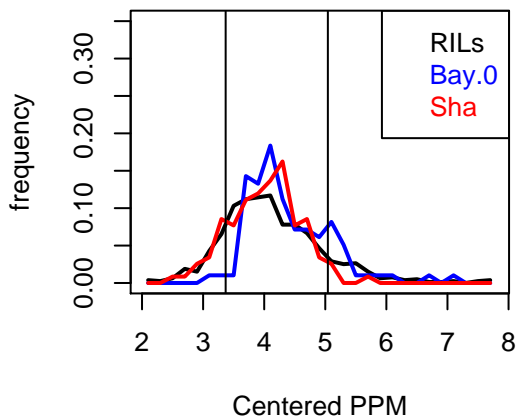**Zn66**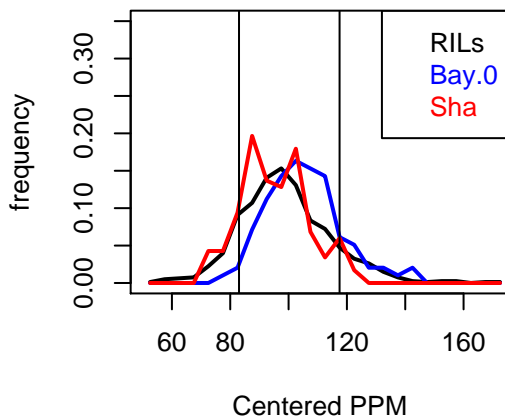**As75**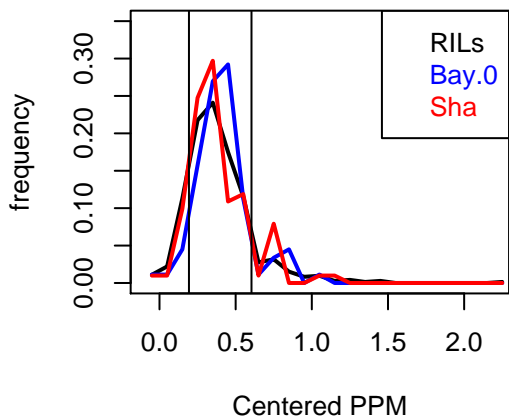**Se82**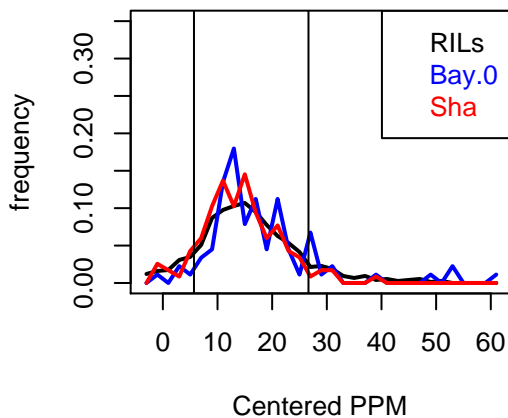

**Rb85**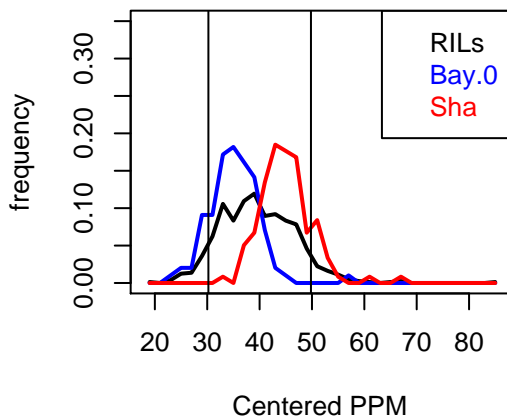**Mo98**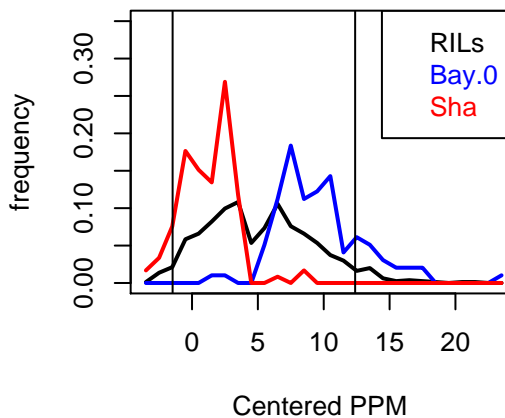**Cd114**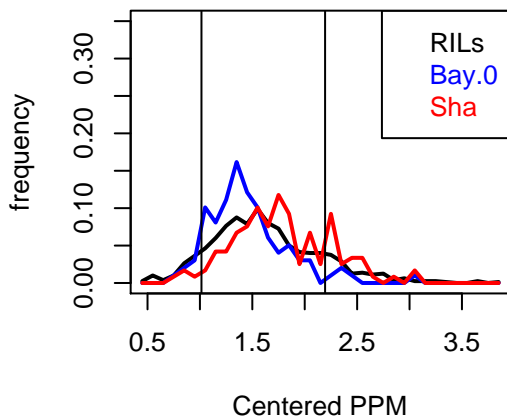

Supplement: File S3 — Frequency plots of parental lines and RILs for each element across the 5 RIL populations. X-axis represents the centered PPM (See Methods) of indicated element. Y-axis indicates frequency of occurrence. Black vertical lines indicate the 95% confidence interval of the parents distribution (i.e. lower parent−1.96 SD (pooled) to higher parent+1.96 SD(pooled)). Comparison BaySha, grown in Promix soil. (0.06 MB PDF) [file pone.0011081.s007.pdf]

**Li7**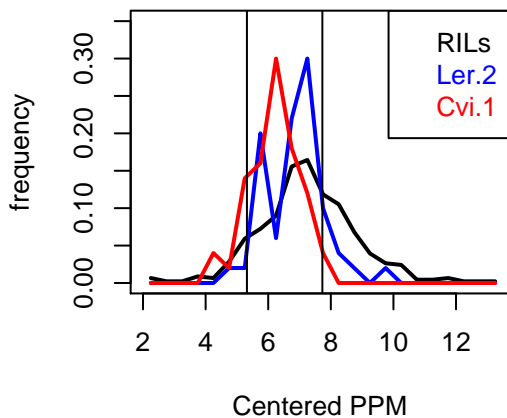**B11**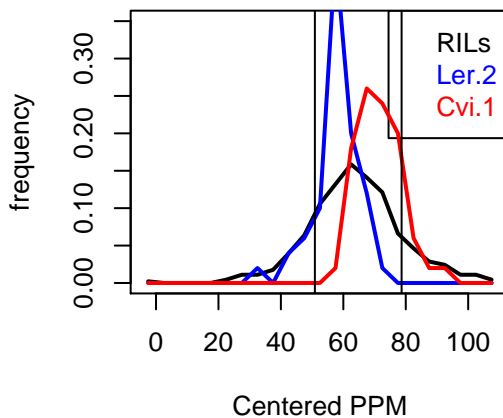**Na23**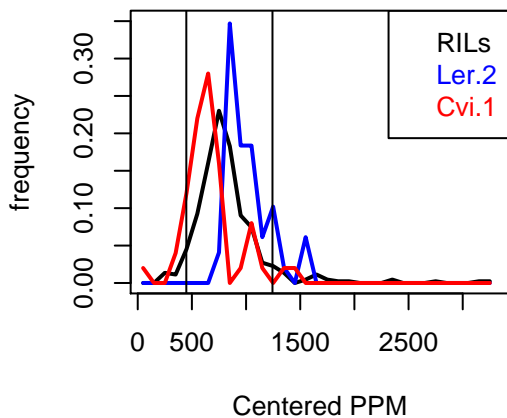**Mg25**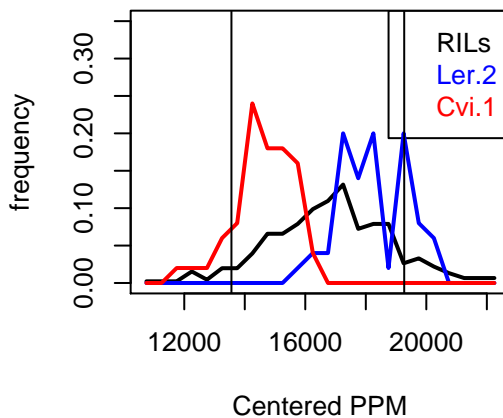

**P31**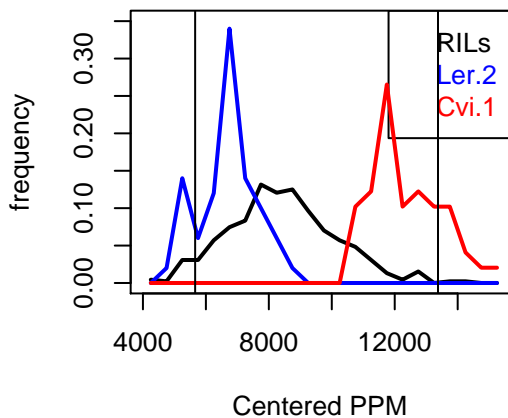**K39**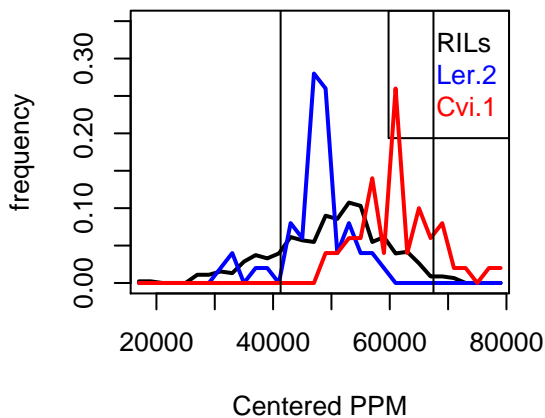**Ca43**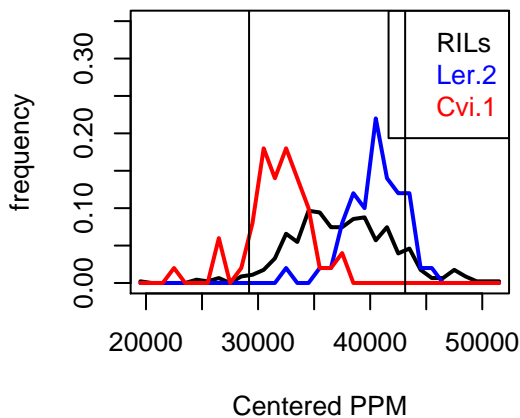**Mn55**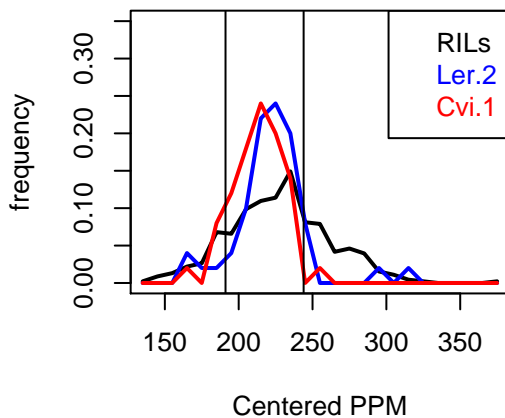

**Fe56**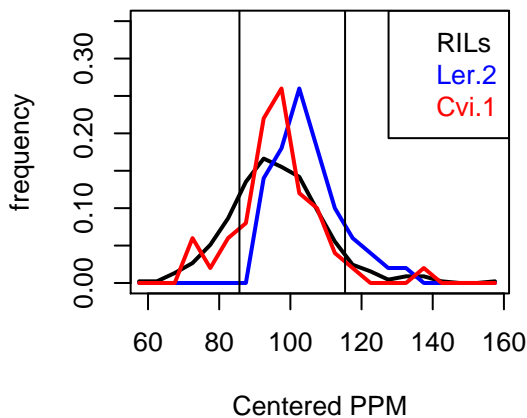**Co59**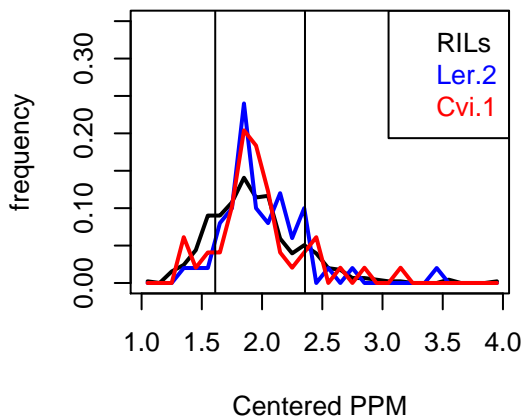**Ni60**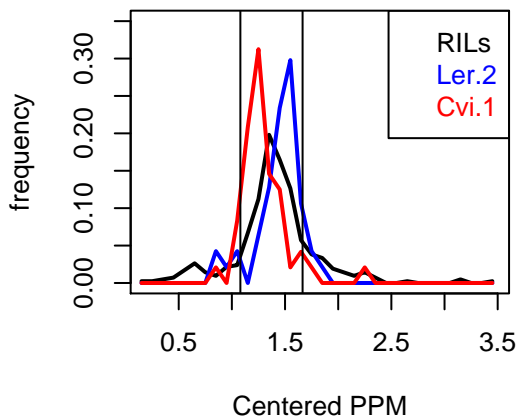**Cu65**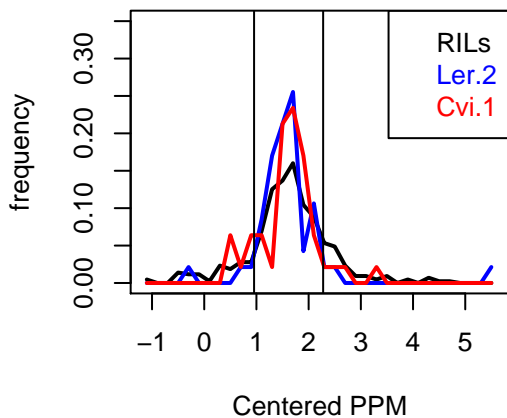

**Zn66**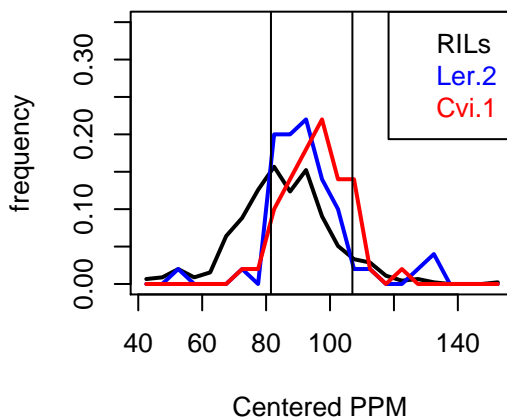**As75**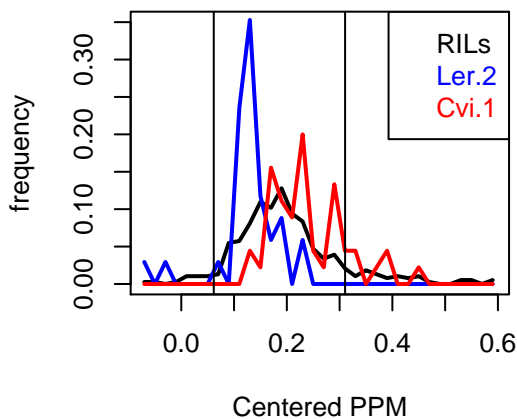**Se77**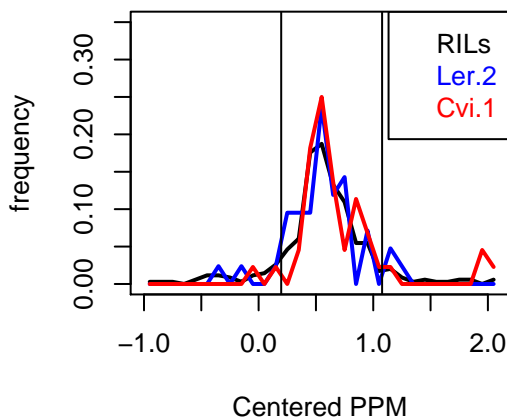**Mo95**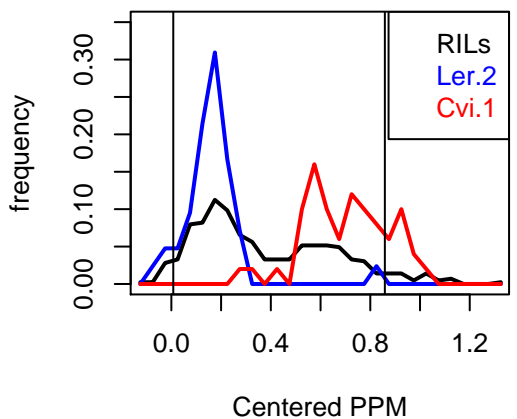

# Cd111

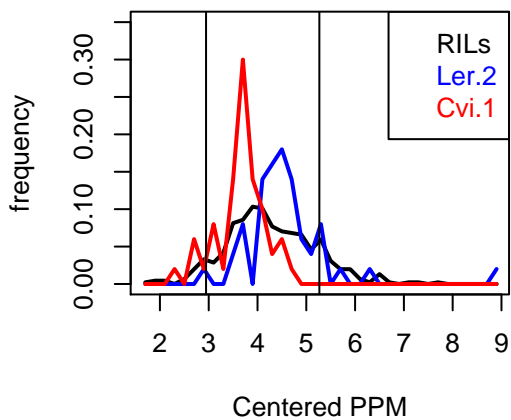

Supplement: File S4 — Frequency plots of parental lines and RILs for each element across the 5 RIL populations. X-axis represents the centered PPM (See Methods) of indicated element. Y-axis indicates frequency of occurrence. Black vertical lines indicate the 95% confidence interval of the parents distribution (i.e. lower parent−1.96 SD (pooled) to higher parent+1.96 SD(pooled)). Comparison of CviLer, high Fe environment. (0.06 MB PDF) [file pone.0011081.s008.pdf]

**Li7**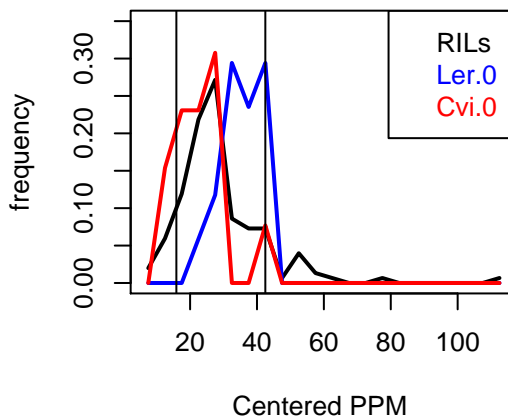**B11**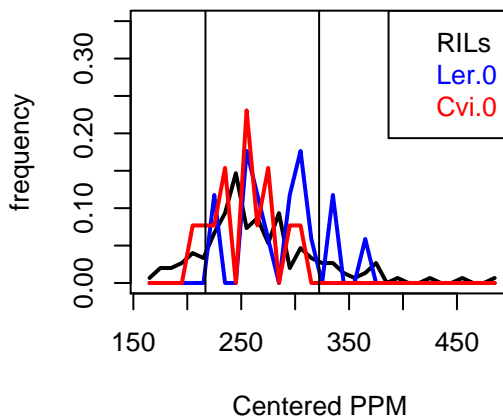**Na23**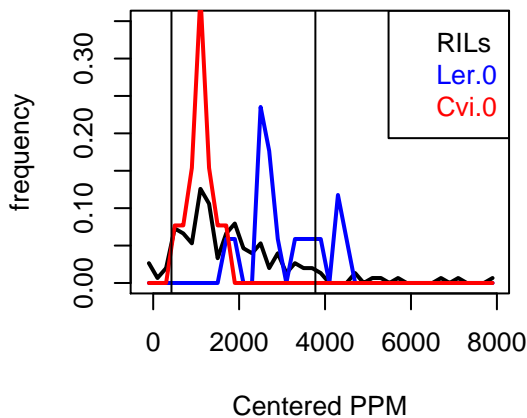**Mg25**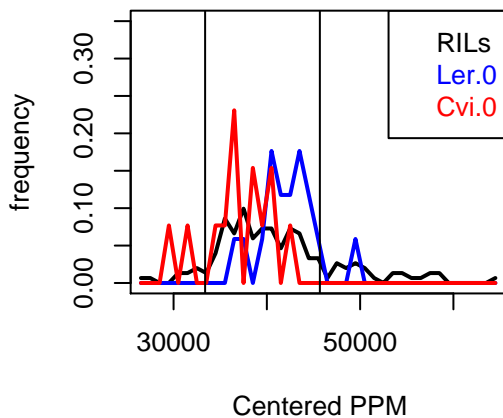

**P31**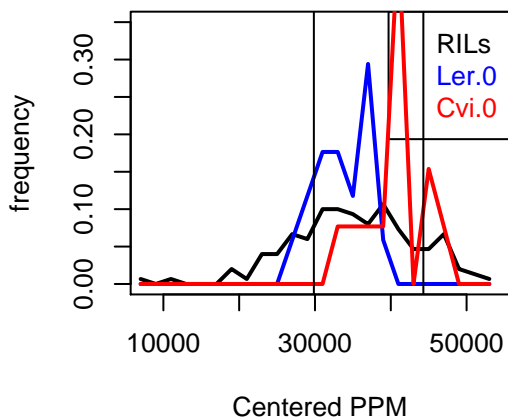**K39**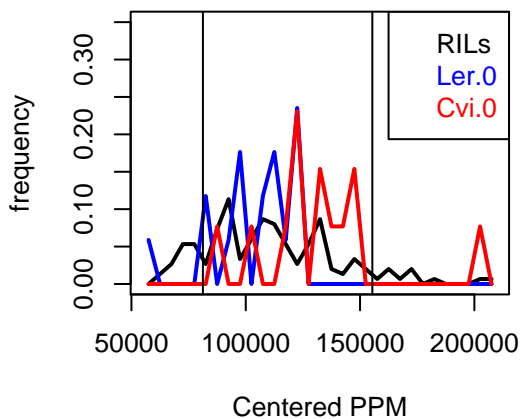**Ca43**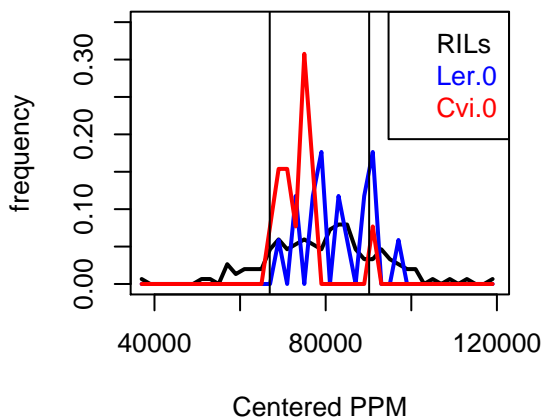**Mn55**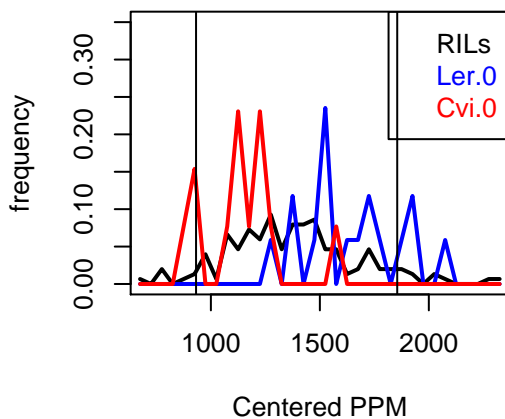

**Fe56**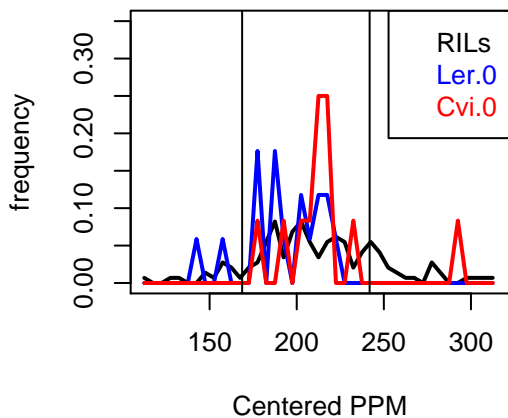**Co59**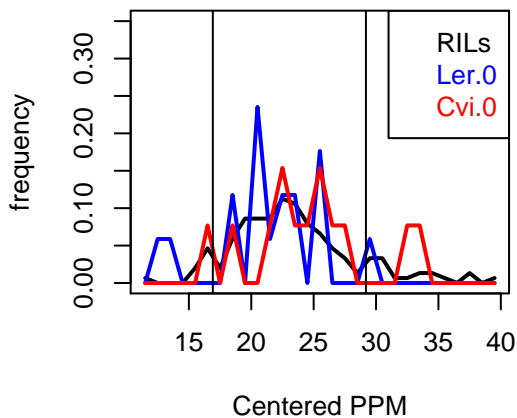**Ni60**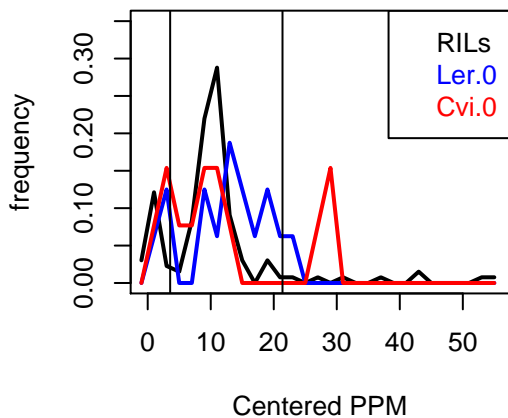**Cu65**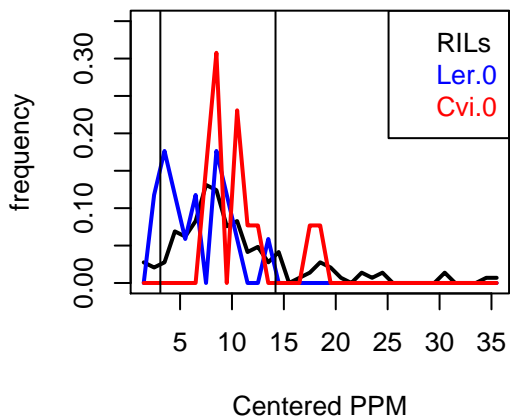

**Zn66**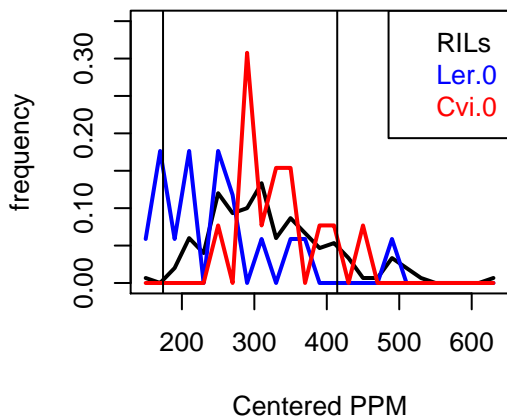**As75**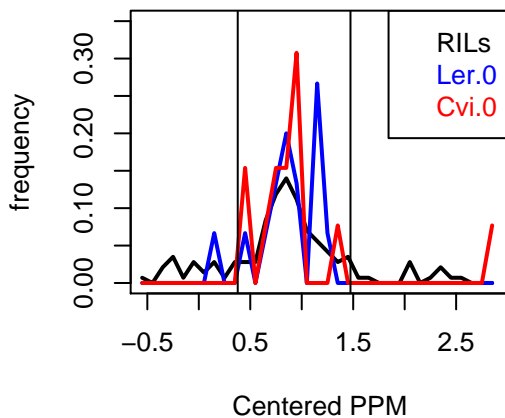**Se77**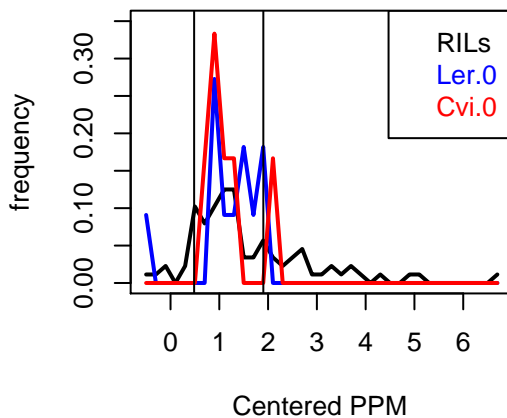**Mo95**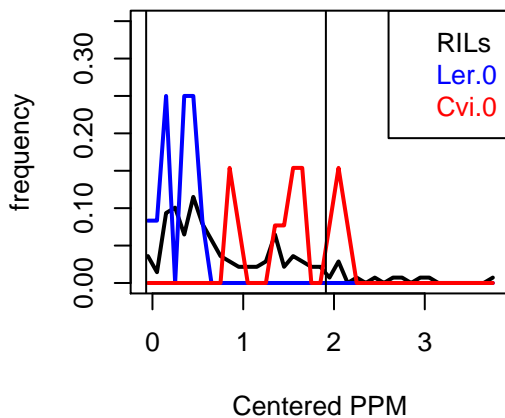

# Cd111

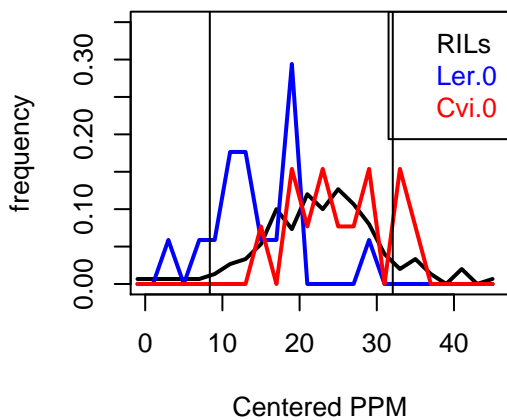

Supplement: File S5 — Frequency plots of parental lines and RILs for each element across the 5 RIL populations. X-axis represents the centered PPM (See Methods) of indicated element. Y-axis indicates frequency of occurrence. Black vertical lines indicate the 95% confidence interval of the parents distribution (i.e. lower parent−1.96 SD (pooled) to higher parent+1.96 SD(pooled)). Comparison of CviLer, low Fe environment. (0.06 MB PDF) [file pone.0011081.s009.pdf]
