# Supplementary material for: Natural Genetic Variation in Selected Populations of Arabidopsis thaliana Is Associated with Ionomic Differences
Source: PLoS One. 2010 Jun 14;5(6):e11081. doi: 10.1371/journal.pone.0011081 (PMC2885407; doi:10.1371/journal.pone.0011081)
Supplement: File S7 — Hoaglands Media Recipe. Modified Hoaglands media used in this study. (0.04 MB DOC) [file pone.0011081.s011.doc]

|  | Hoaglands Mo Type2 | |
| --- | --- | --- |
|  |  |  |
| X | 0.25 |  |
| L | 40 |  |
| NH4H2PO4 (g) | 1.15 |  |
| Ca(NO3)2 (g) | 6.61 |  |
| MgSO4*7H2O (g) | 4.91 |  |
| KNO3 (g) | 6.07 |  |
|  |  |  |
| CuSO4*5H2O (ml) | 10 | note: standard Hoaglands calls for 1 mL |
| ZnSO4*7H2O (ml) | 10 | note: standard Hoaglands calls for 1 mL |
| H2BO3 (ml) | 10 | note: standard Hoaglands calls for 1 mL |
| MnCl2*4H2O (ml) | 10 | note: standard Hoaglands calls for 1 mL |
| MoO3 (ml) | 12.5 | note: standard Hoaglands calls for 1 mL |
|  |  |  |
|  |  |  |
|  |  |  |
| Hoagland's Micronutrients: | 1x stock g/L |  |
| CuSO4*5H2O | 0.075 |  |
| ZnSO4*7H2O | 0.23 |  |
| H3BO3 | 2.844 |  |
| MnCl2*4H2O | 1.01 |  |
| MoO3 | 0.016 |  |
|  |  |  |
| 0.25 x Hoaglands Mo Type2 + 1ml Fe HBED/L - Watering Solution used since 5/27/2005 | | |
